# Elevated N-Glycosylation Contributes to the Cisplatin Resistance of Non-Small Cell Lung Cancer Cells Revealed by Membrane Proteomic and Glycoproteomic Analysis

**DOI:** 10.3389/fphar.2021.805499

**Published:** 2021-12-22

**Authors:** Wenjuan Zeng, Shanshan Zheng, Yonghong Mao, Shisheng Wang, Yi Zhong, Wei Cao, Tao Su, Meng Gong, Jingqiu Cheng, Yong Zhang, Hao Yang

**Affiliations:** ^1^ NHC Key Laboratory of Transplant Engineering and Immunology, Institutes for Systems Genetics, National Clinical Research Center for Geriatrics, West China Hospital, Sichuan University, Chengdu, China; ^2^ Institute of Thoracic Oncology, West China Hospital, Sichuan University, Chengdu, China; ^3^ Sichuan Provincial Engineering Laboratory of Pathology in Clinical Application, West China Hospital, Sichuan University, Chengdu, China

**Keywords:** cisplatin resistance, membrane proteins, N-glycosylation, proteomics, N-glycoproteomics

## Abstract

Chemoresistance is the major restriction on the clinical use of cisplatin. Aberrant changes in protein glycosylation are closely associated with drug resistance. Comprehensive study on the role of protein glycosylation in the development of cisplatin resistance would contribute to precise elucidation of the complicated mechanism of resistance. However, comprehensive characterization of glycosylated proteins remains a big challenge. In this work, we integrated proteomic and N-glycoproteomic workflow to comprehensively characterize the cisplatin resistance-related membrane proteins. Using this method, we found that proteins implicated in cell adhesion, migration, response to drug, and signal transduction were significantly altered in both protein abundance and glycosylation level during the development of cisplatin resistance in the non-small cell lung cancer cell line. Accordingly, the ability of cell migration and invasion was markedly increased in cisplatin-resistant cells, hence intensifying their malignancy. In contrast, the intracellular cisplatin accumulation was significantly reduced in the resistant cells concomitant with the down-regulation of drug uptake channel protein, LRRC8A, and over-expression of drug efflux pump proteins, MRP1 and MRP4. Moreover, the global glycosylation was elevated in the cisplatin-resistant cells. Consequently, inhibition of N-glycosylation reduced cell resistance to cisplatin, whereas promoting the high-mannose or sialylated type of glycosylation enhanced the resistance, suggesting that critical glycosylation type contributes to cisplatin resistance. These results demonstrate the high efficiency of the integrated proteomic and N-glycoproteomic workflow in discovering drug resistance-related targets, and provide new insights into the mechanism of cisplatin resistance.

## Introduction

Cisplatin, a representative anticancer drug, is one of the most widely used chemotherapeutic agents for the clinical treatment of various solid tumors including ovarian, testicular, colorectal, bladder and non-small cell lung cancers (Dilruba and Kalayda, 2016; Rottenberg et al., 2021). Unfortunately, despite the initial effectiveness, the clinical use of cisplatin is dramatically restricted by the development of chemoresistance of cancer cells. The underlying mechanisms of cisplatin resistance are multifactorial and complicated. It has been demonstrated that many pathways are involved in the development of cisplatin resistance, including reduced cellular drug accumulation (Kuo et al., 2012), increased drug detoxification (De Luca et al., 2019), enhanced DNA repair and defective apoptosis signal transduction (Michaud et al., 2009; Ray Chaudhuri et al., 2016). However, these mechanisms are still insufficient to account for the complicated chemoresistance due to the heterogeneous nature of tumor cells and the complexity of cellular responses to cisplatin.

**GRAPHICAL ABSTRACT:**
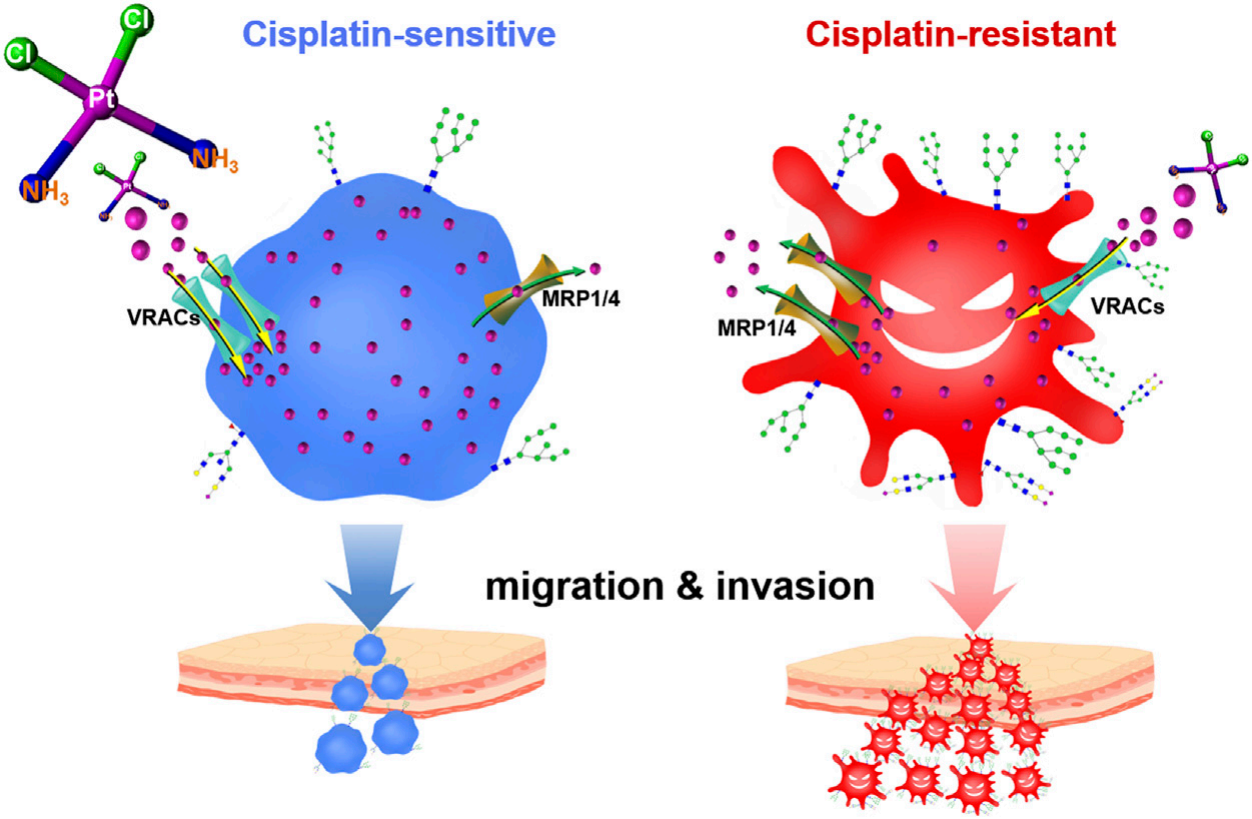


Cell membrane holds multiple membrane carrier proteins (e.g., solute carrier family 22 A1/2/3/4/5, copper transporter 1, volume-regulated anion channels, ATP-binding cassette superfamily), which control the cisplatin uptake and efflux, thereby determining the intracellular drug concentration (Ishida et al., 2002; Wen et al., 2014; Harrach and Ciarimboli, 2015; Planells-Cases et al., 2015; Jia et al., 2021). Importantly, most of these membrane proteins undergo post-translational modifications, particularly with various glycans (Maryon et al., 2007; Voss et al., 2014). Aberrant changes in glycosylation will cause dysfunction of the glycoproteins, consequently giving rise to numerous pathophysiological events, especially those associated with cancer cell growth, invasion, and metastasis (Lau and Dennis, 2008; Christiansen et al., 2014; Zhang et al., 2014). Intriguingly, some recent studies have revealed a correlation between protein glycosylation and chemoresistance, including multidrug resistance in gastric cancer cells, Adriamycin resistance in leukemia cells, and cisplatin resistance in ovarian cancer cells (Li et al., 2013; Sun et al., 2014; Ji et al., 2017; Lin et al., 2020). N-glycans with α2,3-linked sialic structures have been found remarkably higher in cisplatin-resistant ovarian cancer cells and may act as biomarkers to monitor the dynamic process of the acquisition of platinum resistance (Lin et al., 2020). Although protein glycosylation has been expected to provide additional targets for current cancer theranostics (Ferreira et al., 2016; Mereiter et al., 2019; Costa et al., 2020), accurately decoding the site-specific glycosylation profile of the drug resistance-related proteome remains a big challenge (Kolli et al., 2015; Ruhaak et al., 2018), which largely impedes the progress of cancer drug discovery in this field.

Generally, glycoproteomics studies involve the analysis of glycoproteins and site-specific glycans, including the identification of protein, glycopeptide and glycosite, as well as the characterization of site-specific glycan composition and structure (Shah et al., 2015; Gao et al., 2019; Zhang et al., 2020). However, in these previous studies, glycoproteins and glycans were investigated separately. Namely, glycans were released from glycopeptides using N-glycosidase, and either the obtained glycans or deglycopeptides were subjected to mass spectrometry (MS) analysis. Hence, comprehensive information on the role of both glycoproteins and site-specific glycans in chemoresistance remains unavailable. Accordingly, there is still a lack of characterization of cisplatin resistance-associated membrane proteins in both expression and glycosylation profile.

In this work, we integrated a workflow based on high-resolution MS to simultaneously identify glycoprotein, glycopeptide, glycosite, and glycan composition/structure in a single run. Employing this workflow, we analyzed the membrane proteins associated with cisplatin resistance in non-small cell lung cancer cells. We found that critical membrane proteins were altered in abundance and glycosylation during the development of cisplatin resistance, which was related to cell migration, invasion, and cisplatin accumulation. Our results demonstrated that elevated high-mannose or sialylated type of glycosylation attenuated the sensitivity of non-small cell lung cancer cells to cisplatin and contributed to the resistant phenotype. These findings provide new insights into the mechanisms underlying cisplatin resistance.

## Materials and Methods

### Materials and Chemicals

Cisplatin, dithiothreitol (DTT), iodoacetamide (IAA), formic acid (FA), trifluoroacetic acid (TFA), concanavalin A (Con A) and horseradish peroxidase (HRP) were obtained from Sigma (St. Louis, MO, United States). Acetonitrile (ACN) was purchased from Merck (Darmstadt, Germany). Sequencing-grade trypsin was obtained from Enzyme and Spectrum (Beijing, China). Minute™ Plasma Membrane Protein Isolation and Cell Fractionation Kit (SM-005) was obtained from Invent Biotechnologies (Eden Prairie, MN, United States). The rabbit monoclonal anti-human Na/K ATPase antibody (ab76020), anti-human Lamin-B1 antibody (ab133741), anti-human Tomm20 antibody (ab186735), anti-human α-1,2-mannosidase ІA (MAN1A1) antibody (ab140613), anti-human sialidase-1 (Neu1) antibody (ab197020), anti-human leucine-rich repeat-containing 8A (LRRC8A) antibody (ab157489), and anti-human multidrug resistance-associated protein 1 (MRP1) antibody (ab180960) were purchased from Abcam (Cambridge, MA, United States). Rabbit monoclonal anti-human β-actin antibody (AC026) was purchased from Abclonal (Wuhan, China), and goat anti-rabbit horseradish peroxidase-conjugated secondary antibody (511,203) was purchased from Zen Bioscience (Chengdu, China). The glycosylation inhibitors, Tunicamycin and Kifunensine, were obtained from Abcam (Cambridge, MA, United States), Oseltamivir phosphate from SelleckChem (Houston, TX, United States), and Castanospermine from MedChemExpress (United States). Zwitterionic hydrophilic interaction liquid chromatography (Zic-HILIC) materials were obtained from Fresh Bioscience (Shanghai, China). Deionized water was prepared using a Milli-Q system (Millipore, Bedford, MA, United States). All other chemicals and reagents of the best available grade were purchased from Sigma-Aldrich or Thermo Fisher Scientific.

### Cell Culture and Drug Treatment

Human non-small cell lung cancer cell line, A549, and its cisplatin-resistant counterpart, A549/DDP, were obtained from KeyGen Biotech (Nanjing, China) with the authentication by short tandem repeat (STR) detection. The cisplatin-resistance of A549/DDP cells was acquired by continuously exposing A549 cells to cisplatin of progressive concentrations until the final concentration reached 1.0 μg/ml. A549 cells were cultured in RPMI-1640 (Gibco) supplemented with 10% fetal bovine serum (FBS, Gibco) and 100 U/ml penicillin-streptomycin (Hyclone). The same medium with 1.0 μg/ml cisplatin was used to culture A549/DDP cells. However, to avoid the influence of cisplatin, A549/DDP cells were cultured in a drug-free medium for 3 days prior to subsequent experimentation. All cells were grown at 5% CO_2_ in a humidified incubator at 37°C. Cisplatin was dissolved in phosphate buffered saline (PBS) to prepare a 1.0 mM stock solution and further diluted with culture medium to desired concentration.

### Cell Fractionation, Protein Extraction, and Protein Characterization

A549 and A549/DDP cells were washed and harvested using a cell scraper when reaching 80% confluence, followed by wash and centrifugation with PBS for three times. Cells were fractionated into four parts, the cell membrane (CM), organelle (CO), nucleus (CN), and cytosol (CC) using the Minute™ Plasma Membrane Protein Isolation and Cell Fractionation Kit following the manufacturer’s instructions. Intact cells and the obtained CM, CO and CN fractions were lysed using UA buffer (8 M urea, 0.1 M Tris, pH 8.5) with sonication at 4°C for protein extraction. The protein concentration was determined using bicinchoninic acid assay (BCA) kit (Thermo Fisher Scientific, United States). Proteins from the CM, CO, CN, and CC fraction, along with the whole cell lysate (WP) were boiled with gel-loading buffer, and then loaded onto a 4–20% gradient gel (Genscript, Nanjing, China) for sodium dodecyl sulfate polyacrylamide gel electrophoresis (SDS-PAGE). The gel was stained with Coomassie brilliant blue for 10 min, washed with water overnight, and imaged. For Western blot (WB) analysis, the separated proteins were transferred to a polyvinylidene fluoride (PVDF) membrane (Millipore, United States). The membrane was blocked in 5% skimmed milk in Tris-buffered saline (TBS) with 0.1% Tween20 (TBST) at room temperature for 1 h, and then incubated with primary antibodies of human Na/K ATPase, Lamin-B1, Tomm20, and β-actin in appropriate dilutions at 4°C overnight. The blots were washed four times with TBST for 5 min each and incubated with HRP-conjugated secondary antibody at room temperature for 1 h. After being washed with TBST, the protein bands were visualized by enhanced chemiluminescence (ECL, Thermo Fisher Scientific, United States) and imaged using an image analyzer (Tanon 4600SF, China).

### Sample Preparation for MS Analysis

Cell membrane proteins were prepared in three biological replicates for each cell line from the cell culture step. The membrane proteins were prepared for MS analysis using a modified filter-aided sample preparation (FASP) protocol. In brief, 100 μg of proteins were loaded onto a 30-kDa filter and centrifuged at 13,000 g for 15 min. After being reduced by DTT (20 mM) for 4 h at 37°C and alkylated with IAA (50 mM) for 30 min at 25°C in the dark, the protein mixture was washed once with 200 μl UA buffer and three times with 200 μl of 50 mM ammonium bicarbonate by centrifugation at 13,000 g for 15 min at room temperature. Then 200 μl of 50 mM ammonium bicarbonate containing 2.0 μg trypsin was added to each filter and incubated for 16 h at 37°C. Finally, the filter tubes were washed three times with 100 μl water by centrifugation at 13,000 g for 10 min. The concentration of these collected peptides was determined using a peptide assay kit (Thermo Fisher Scientific, United States) based on the absorbance measured at 480 nm. The peptide mixtures were freeze-dried and then stored at −80°C.

### Enrichment of Intact N-Glycopeptides

Intact N-glycopeptides were enriched by the Zic-HILIC method. Specifically, the peptide mixtures were re-dissolved in 100 μl equilibration buffer (80% ACN/0.2% TFA), and 2 mg processed Zic-HILIC materials were added and rotated for 2 h at 37°C. Finally, the mixture was transferred to a 200-μl pipette tip packed with a C8 membrane and washed twice with 70 μl equilibration buffer. Intact N*-*glycopeptides were then eluted three times with 70 μl elution buffer (0.1% TFA in water) and were freeze-dried for further analysis.

### LC-MS/MS Analysis

All samples were analyzed by LC-MS/MS in the data-dependent acquisition mode using an Orbitrap FusionLumos mass spectrometer (Thermo Fisher Scientific). In brief, peptides and intact N-glycopeptides of membrane proteins were dissolved in water with 0.1% FA and separated on a column (ReproSil-Pur C18-AQ, 1.9, 75 μm inner diameter, length 20 cm; Dr Maisch) over a 78-min gradient (solvent A, 0.1% FA in water; solvent B, 0.1% FA in 80% ACN) at a flow rate of 300 nl/min (0–8 min, 5–8% B; 8–58 min, 8–22% B; 58–70 min, 22–32% B; 70–71 min, 32–90% B; and 71–78 min, 90% B).

MS parameters for the detection of peptides were as follows. 1) MS1: orbitrap resolution = 120,000; scan range (*m/z*) = 350–1,550; RF lens = 30%; AGC target = 1.0 e^6^; maximum injection time = 50 ms; included charge state = 2–6; exclusion duration = 15 s; exclusion after n times, *n* = 1; each selected precursor ion was subjected to one high-energy collision dissociation MS/MS (HCD-MS/MS); 2) MS2: isolation window (*m/z*) = 2.0; HCD collision energy = 35%; detector type = orbitrap; orbitrap resolution = 15,000; first mass (*m/z*) = 120; AGC target = 5.0 e^4^; maximum injection time = 80 ms.

MS parameters for the detection of intact N-glycopeptides were as follows. 1) MS1: orbitrap resolution = 120,000; scan range (*m/z*) = 800–2000; RF lens = 30%; AGC target = 2.0 e^5^; maximum injection time = 100 ms; included charge state = 2–6; exclusion duration = 15 s; exclusion after n times, *n* = 1; each selected precursor ion was subjected to one stepped collision energy high-energy collision dissociation MS/MS (SCE-HCD-MS/MS); 2) MS2: isolation window (*m/z*) = 2.0; detector type = orbitrap; orbitrap resolution = 15,000; first mass (*m/z*) = 120; AGC target = 5.0 e^5^; maximum injection time = 250 ms; HCD collision energy = 30%; stepped collision energy mode on, energy difference of ± 10% (20–30–40%).

### Data Processing

For the identification and quantification of cell membrane protein, the raw peptide data files were searched against the human uniprot database (v. 2020_08; 20,368 entries) with MaxQuant (v. 1.5.3.8; Max Planck Gesellschaft, Munich, Germany). Label-free quantification (LFQ) analysis of the proteins was performed. Two missed cleavage sites were allowed for trypsin digestion. Cysteine carbamidomethylation (+57.02 Da) was set as the fixed modification. Oxidation of methionine (+15.99 Da), deamidation of asparagine (+0.98 Da), and acetylation of the protein N-terminal (+42.01 Da) were set as variable modifications. Match between runs was used for the alignment of retention time across the six individual LC-MS/MS runs. The false discovery rates (FDR) of the peptide spectrum matches (PSMs) and proteins were set to <1%.

For the identification of the intact N-glycopeptides, the raw data files were searched using Byonic software (version 3.6.1, Protein Metrics, Inc.), with the mass tolerance for precursors and fragment ions set at ± 6 ppm and ±20 ppm, respectively. Two missed cleavage sites were allowed for trypsin digestion. The fixed modification was carbamidomethyl (C), and variable modifications included oxidation (M), acetylation (protein N-term), and deamidation (N). In addition, 182 human N-glycans were specified as N-glycan modifications. All other parameters were set as the default values, and the protein groups were filtered to a 1% FDR based on the number of hits obtained for searches against these databases. Stricter quality control methods for intact N-glycopeptide identification were implemented, requiring a score of no less than 300 and identification of at least six amino acids. Furthermore, all of these PSMs and glycopeptide-spectrum matches (GPSMs) were examined manually and filtered using the following criteria: PSMs were accepted if there were at least 3 b/y ions identified in the peptide backbone, and GPSMs were accepted if there were at least three glycan oxonium ions identified and at least three 3 b/y ions identified in the peptide backbone.

### Bioinformatics Analysis

We performed the bioinformatics analysis using an R language-based in-house freely available platform called “Wu Kong” (https://wkomics.omicsolution.com/wkomics/main/) (Wang et al., 2020). For quantitative analysis of the proteomics data, the LFQ intensities of cell membrane proteins from A549 and A549/DDP cells were extracted from the MaxQuant result file to represent the expression level of corresponding protein in six samples. Proteins with the number of missing LFQ intensity accounting for 50% or more across all samples were filtered, after which the intensity was normalized to the median and then the missing value was filled using *k*-nearest neighbors (KNN) imputation. To get precise quantitation results, proteins with a coefficient of variation of the intensities larger than 0.3 were filtered. After filtering, a 1,360 × 6 protein expression matrix was generated for subsequent statistical analysis. Unpaired Student’s *t*-test was performed to screen the differentially expressed proteins (DEPs) between A549 cell membrane (ACM) and A549/DDP cells membrane (DCM) with a Benjamini-Hochberg (BH) adjusted *p* value <0.05 and a fold change >2.00. Biological function of the DEPs was analyzed through Gene Ontology (GO) enrichment analysis and Kyoto Encyclopedia of Genes and Genomes (KEGG) pathway enrichment analysis using the “Wu Kong” platform. For quantitative analysis of the intact N-glycopeptides, the intensities of intact N-glycopeptides were extracted from the Byonic result files to represent the abundance of corresponding intact N-glycopeptides in the six samples. Glycopeptides having more than 50% missing data were excluded. The intensity was normalized to the median and then the missing values were filled using KNN imputation. Moreover, glycopeptides with a coefficient of variation of the intensities larger than 0.5 were filtered. Unpaired Student’s *t*-test was performed to screen the differentially expressed glycopeptides (DEGs) between ACM and DCM groups with a BH adjusted *p* value <0.05 and a fold change >2.00. In particular, we reserved the intact N-glycopeptides that were identified in all the three biological replicates within one group while completely missing in all replicates of the other.

### WB Analysis and Glycoprotein Detection

Proteins extracted from ACM and DCM, or the whole cell lysate of A549 and A549/DDP were transferred to PVDF membrane after SDS-PAGE separation. For the detection of high-mannose glycosylated protein, the membrane was blocked with TBST at room temperature for 1 h, followed by the incubation with 25 μg/ml Con A in TBST containing 1 mM CaCl_2_ and 1 mM MnCl_2_ (TBSTS) at 4°C overnight. The blots were washed four times with TBSTS for 5 min each, and then incubated with 0.5 μg/ml HRP at room temperature for 1 h. After being washed with TBSTS, the protein bands were visualized by ECL and imaged using an image analyzer (Tanon 4600SF, China). For the detection of MAN1A1, Neu1, LRRC8A and MRP1 proteins, the PVDF membrane was blocked with 5% skimmed milk in TBST at room temperature for 1 h, followed by the incubation with corresponding primary antibodies in appropriate dilutions at 4°C overnight. The blots were washed with TBST and incubated with HRP-conjugated secondary antibody at room temperature for 1 h. The protein bands were also visualized by ECL and imaged by the image analyzer. Na/K ATPase was used as the internal standard for the detection of cell membrane proteins LRRC8A, MRP1 and the global high-mannose glycosylated membrane proteins. In particular, for the detection of MRP1, the samples were not boiled before loading onto the SDS-PAGE gel to reduce protein aggregation. β-actin was used as the internal standard for the detection of MAN1A1 and Neu1 from the whole cell lysates.

### Inductively Coupled Plasma Mass Spectrometry (ICP-MS) Detection

A549 and A549/DDP cells with a density of 1 × 10^6^ were cultured for 24 h, and then treated with 30 μM cisplatin for 24 h. After rinsing with PBS twice, the cells were collected, further washed three times with PBS, and counted. Subsequently, the cells were lysed with 250 μl of 4% SDS via repeated freeze-thaw cycles in liquid nitrogen followed by sonication. Then, the cell lysate was diluted to 1 ml with water, filtered through a 0.22 μm filter, and injected into the ICP mass spectrometer (7,700, Agilent, United States) to determine the concentration of platinum (Pt). A standard curve was generated using a certified platinum standard solution. The amount of Pt in cells was calculated from the standard curve and reported in nanogram per million cells (Pt ng/10E6 cells). All samples were prepared and detected in quadruplicate to obtain the average and standard deviation (SD).

### Methyl Thiazolyl Tetrazolium (MTT) Assay

Cytotoxicity of cisplatin alone or the co-administration of cisplatin and glycosylation inhibitors was evaluated in A549 and A549/DDP cells using MTT assay. Cells (5 × 10^3^) were seeded in a 96-well plate and incubated at 37°C overnight. For the individual administration of cisplatin or the glycosylation inhibitor (Tunicamycin, Kifunensine, Castanospermine or Oseltamivir phosphate), 200 μl of culture medium with different concentrations of cisplatin (1–100 μM) or glycosylation inhibitor (2–200 μM) were added to each well followed by incubation for 48 h. For the co-administration of cisplatin and glycosylation inhibitor, 200 μl of medium with inhibitor and different concentrations of cisplatin were added to each well and incubated for 48 h. The medium was removed, and 100 μl of 0.5 mg/ml solution of MTT in RPMI-1640 medium were added to each well and incubated for another 3.5 h. Then, the medium was fully aspirated, and 150 μl DMSO were added to dissolve the purple formazan. Absorbance at 562 nm was determined, and cell viability or death was calculated.

### Transwell Migration and Invasion Assays

Cell migration assay was conducted using a 24-well Transwell chamber with a polycarbonate membrane filter of 8 μm pore size (Corning). A549 or A549/DDP cells (5 × 10^5^) suspended in 200 μl serum-free RPMI-1640 media were seeded in the upper compartment of the chamber, and 500 μl RPMI-1640 with 2.5% FBS were added to the lower compartment of the chamber. Cells were incubated at 37°C with 5% CO_2_ for 24 h to allow migration, after which the cells were fixed with 4% paraformaldehyde for 20 min and stained with hematoxylin for 20 min at room temperature. After being washed three times with PBS, the non-migrating cells in the upper chamber were carefully removed using a cotton swab. The migrated cells on the lower surface of the membrane were imaged and photographed with an inverted microscope (Olympus IX83) at 200× magnification in five randomly selected visual fields, and the migrated cells were counted using Image J software with manual inspection. Each assay was performed in triplicate, and the number of migrated cells was shown as means ± SD. Cell invasion assay was performed similarly, except that the FBS concentration in the lower compartment of the chamber was increased to 8%, and the incubation time was extended to 48 h.

### Molecular Modeling

The molecular model of the glycosylated human volume-regulated anion channel (VRAC) protein was constructed using the Sybyl X 2.0 program (Tripos Inc.) running on a Dual-core Intel(R) E5300 CPU, 2.60 GHz, 4 GB RAM, under the Windows 10 operating system. Crystal structure of the human VRAC protein was obtained from the Protein Data Bank (PDB code: 6DJB). The oligosaccharide was constructed using Sybyl 2.0 program and docked onto the protein’s binding site (Asn66 at Chain A) via the formation of C-N glycosidic bond between the C-1 atom of the terminal N-acetylglucosamine and the N atom of the sidechain amide of the asparagine residue. The initial covalent bond distance of C-N was set to 1.4 Å, and all the hydrogen atoms were added to define the correct configuration and tautomeric states. Then Amber7 FF99 charges were added to the biopolymer and all the atoms were set as Amber7 FF99 type, followed by stepwise energy minimization under standard set parameters using the Amber7 FF99 force field with the Powell energy minimization algorithm, a distance dependent dielectric function, and current charges with the 0.05 kcal·mol^−1^ constringent energy gradient to generate the molecular model. The final schematic diagrams were created using PyMOL. The calculation of the solvent accessible area (SAA, Å^2^) of the NH_2_ group on the sidechain of Asn66 residue before and after glycosylation was performed using the VEGA ZZ module of PyMOL program.

## Results and Discussion

### Workflow for the Integrated Proteome and Glycoproteome Analytical Method

**FIGURE 1:**
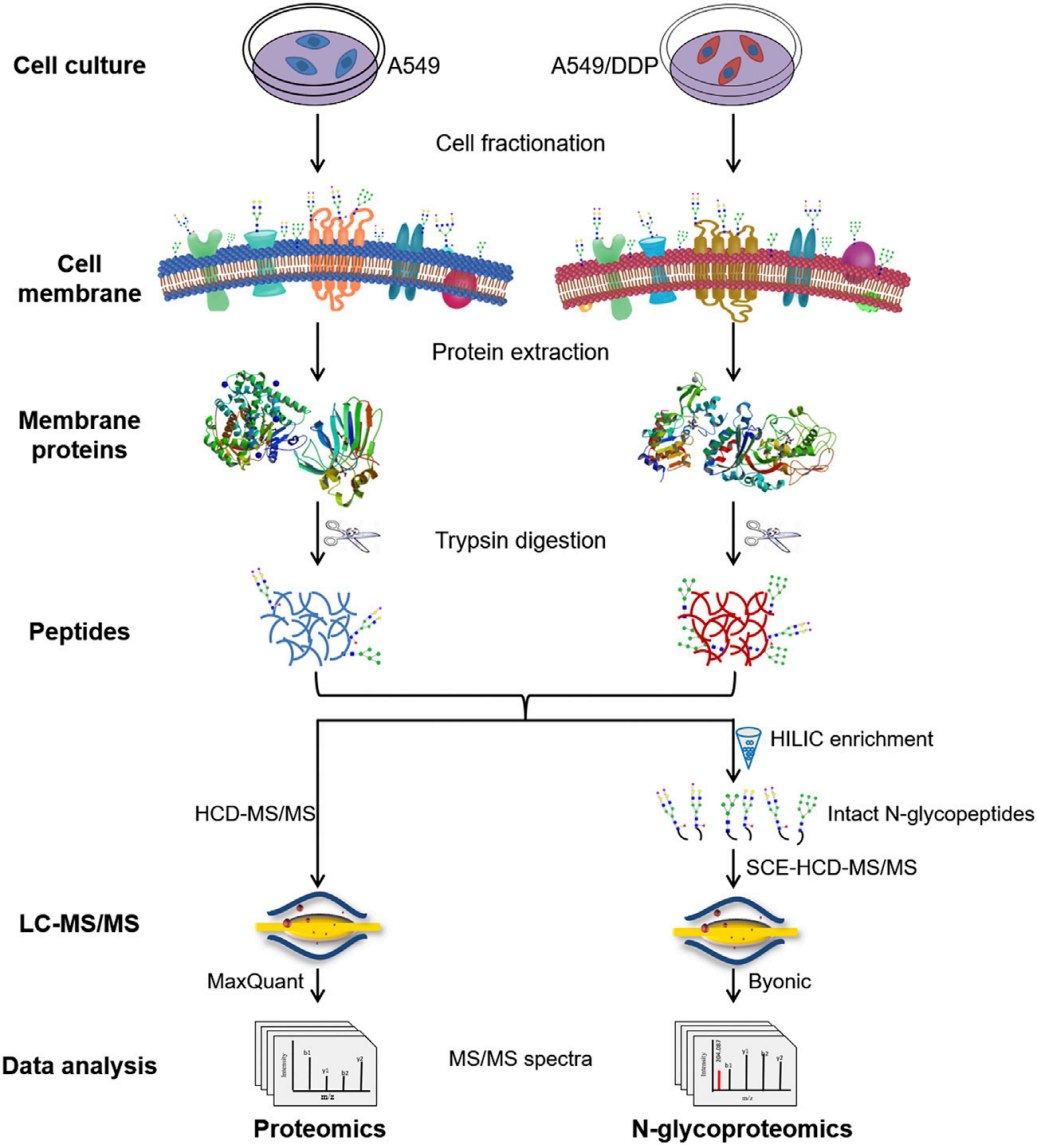
Schematic illustration of the workflow for the integrated proteomic and N-glycoproteomic method designed to comprehensively profile cisplatin-resistance-related cell membrane proteins, N-glycoproteins, and N-glycans of the non-small cell lung cancer cell line.

The integrated proteome and glycoproteome analytical workflow was composed of four major steps, including cell culture and fractionation, membrane protein extraction and digestion, proteomic analysis, and N-glycoproteomic analysis (Figure 1).

The cisplatin-resistant A549/DDP cell line was established by continuously exposing the parental A549 cell line to cisplatin of increasing concentrations till 1.0 μg/ml. The resistance factor was determined to be 8.36 by measuring the cytotoxicity of cisplatin to A549 and A549/DDP cells (Supplementary Figure S1), verifying the cisplatin-resistance of A549/DDP cells. A549 and A549/DDP cell fractionation was performed using a membrane extraction kit. Cell membrane proteins, the proteins from other fractions, and the whole cell lysate were analyzed by SDS-PAGE and WB to examine the purity of membrane proteins (Supplementary Figures S2A,B). WB analysis showed that the cell membrane marker, Na/K ATPase, was significantly enriched in the cell membrane fraction, validating the high purity of the obtained cell membrane. Then, the membrane proteins were denatured, reduced, alkylated and digested into peptides, which were further divided in half for subsequent proteomic and N-glycoproteomic analysis. For proteomic analysis, the peptides were detected by HCD-MS/MS, and quantified by LFQ method. For N-glycoproteomic analysis, the intact N-glycopeptides were enriched by HILIC beads, and then directly analyzed by SCE-HCD-MS/MS with flexible collision energy settings (20–30–40%). In this manner, abundant and informative fragment ions from both the glycan and the peptide can be produced and detected within one spectrum, allowing the simultaneous identification of glycoproteins, glycopeptides, glycosites, and glycan composition/structure in a single run. This integrated method is able to simultaneously analyze membrane proteins and N-glycoproteins as well as N-glycans by intact glycopeptide analysis in a single process, making it suitable to comprehensively characterize the alterations in both abundance and glycosylation of membrane proteins involved in the development of cisplatin-resistance.

### Proteomic Screening of the Resistance-Related Cell Membrane Proteins

**FIGURE 2:**
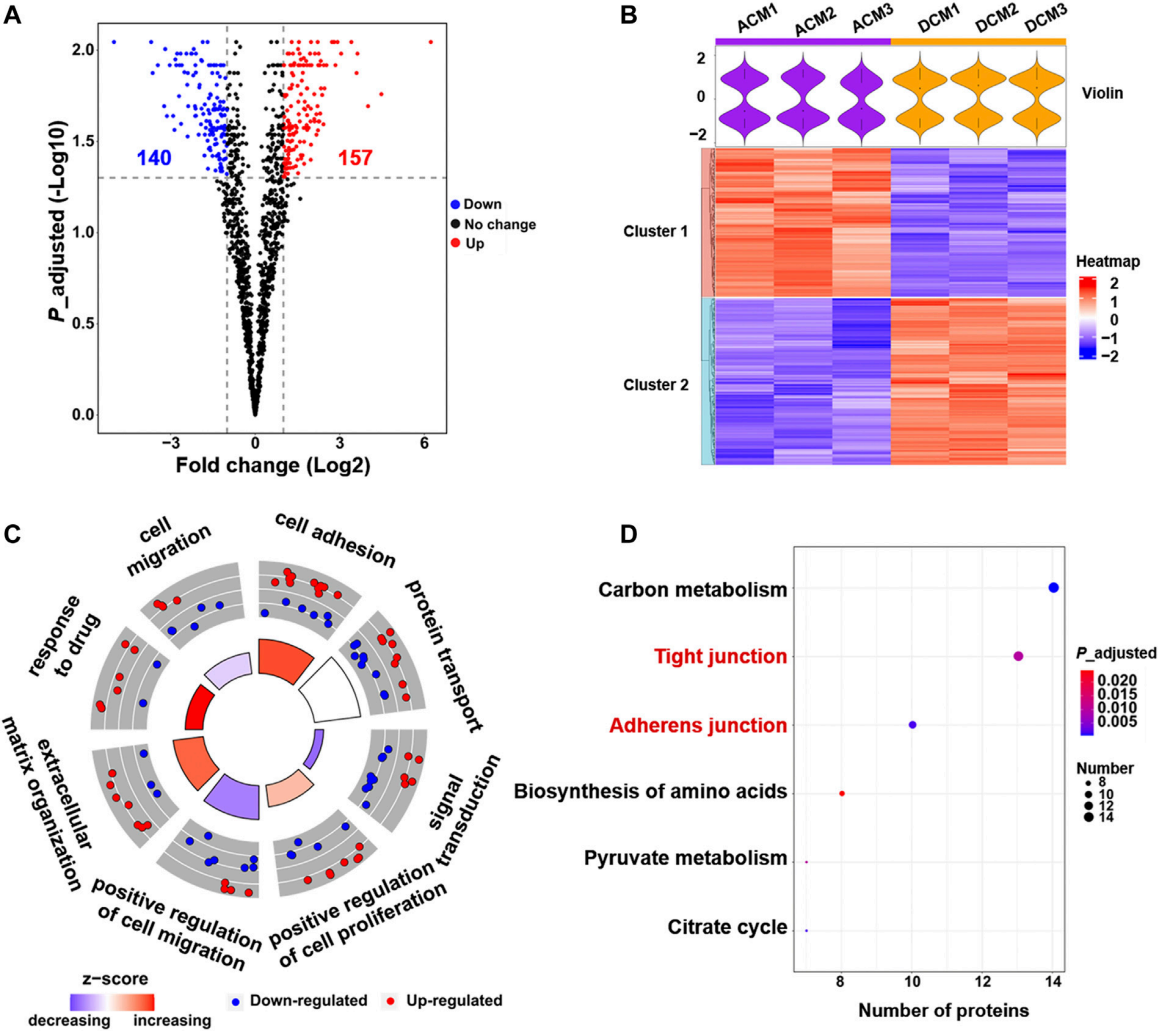
Quantitative proteomic analysis of cell membrane proteins. **(A)** Volcano plot of proteomic data constructed using fold changes (DCM/ACM) and adjusted *p* values. Red dots: significantly up-regulated proteins (fold change >2.00, *p* < 0.05). Blue dots: significantly down-regulated proteins (fold change <0.50, *p* < 0.05). Black dots: proteins with no significant changes. **(B)** Heat map generated from the normalized intensity of the 297 DEPs across ACM and DCM groups. The upper violin plot shows the intensity distribution of proteins in each sample. **(C)** GOCircle plot of the top eight enriched biological processes of the 297 DEPs. The up-regulated (red dots) and down-regulated (blue dots) proteins in each process are distributed in the outer circle of the plot. The inner circle displays the z-score, calculated as the number of up-regulated proteins minus the number of down-regulated proteins divided by the square root of the total count. The larger z-score represents more up-regulated proteins enriched in the process. **(D)** Top six enriched KEGG pathways of the DEPs.

Comparative proteomic analysis was performed to characterize the abundance changes of membrane proteins in A549/DDP cells compared with parental A549 cells. The experiments were conducted in triplicate and a total of 2,906 proteins were identified. GO enrichment analysis showed that these proteins were mainly enriched in the membrane component (Supplementary Figure S2C), further confirming the purity of the extracted proteins. To identify the proteins associated with cisplatin resistance, we carried out the quantitative proteomic analysis based on LFQ method, and the quantitation results were shown as the fold change of DCM/ACM. A total of 297 cell membrane DEPs were screened out, among which 157 proteins were up-regulated with a fold change >2.00, and 140 were down-regulated with a fold change <0.50 (Figure 2A). The abundance of these membrane proteins was significantly altered in drug-resistant A549/DDP cells, suggesting their involvement in the development of cisplatin resistance. The DEPs could be classified into two distinct clusters between ACM and DCM groups, and the normalized intensities of the same protein were similar in the three biological replicates, indicating good reproducibility and reliability of the results (Figure 2B).

To explore the functions of these DEPs, the biological processes and pathways they participate in were analyzed through GO enrichment analysis and KEGG pathway enrichment analysis. The GO analysis results were further integrated with the fold changes of these proteins to generate the GOCircle plot (Figure 2C), which could clearly present the major enriched biological processes and the composition of up- and down-regulated proteins in each process, facilitating the comparison of biological functions amongst these DEPs. As shown in Figure 2C, the biological processes involving more up-regulated proteins mainly included cell adhesion and response to drug, which were expected to enhance cell adhesion and the tolerance to cisplatin, thereby contributing to drug resistance. In contrast, most of the down-regulated membrane proteins were implicated in signal transduction and cell migration, implying the deficient signal transduction and reduced migration of A549/DDP cells.

GO analysis revealed that cell adhesion and response to drug were enhanced, while signal transduction and cell migration appeared to be impaired in the cisplatin-resistant cells (Figure 2C, Supplementary Figure S3). Moreover, KEGG pathway analysis indicated that the 297 DEPs mainly functioned in the cell junction and metabolism pathways (Figure 2D). In particular, the up-regulated proteins were concentrated on the tight junction and adherens junction pathways (Supplementary Figures S4, S5), which supported the result of GO analysis that cell adhesion was increased in the resistant cells. Altogether, proteomic screening highlights that some critical membrane proteins implicated in cell adhesion, response to drug, cell migration and some signal pathways are altered in the abundance during the development of cisplatin resistance.

### N-Glycoproteomic Profiling of Resistance-Related Membrane Glycoproteins and Glycans

N-glycoproteomic analysis was employed to comprehensively study the alteration of membrane protein glycosylation in multiple aspects, including proteins, glycopeptides, glycosites, and glycans. By virtue of SCE-HCD-MS/MS, simultaneous identification of N-glycoproteins and N-glycans can be achieved in a single run. N-glycoproteome profiling of whole cell lysate was also performed and compared with that of membrane proteins. The identified intact N-glycopeptides, N-glycopeptides (peptide backbones without glycans), and N-glycosites were significantly elevated in the membrane protein groups (Supplementary Figure S6), which contributed to the identification of N-glycoproteins with higher confidence.

**FIGURE 3:**
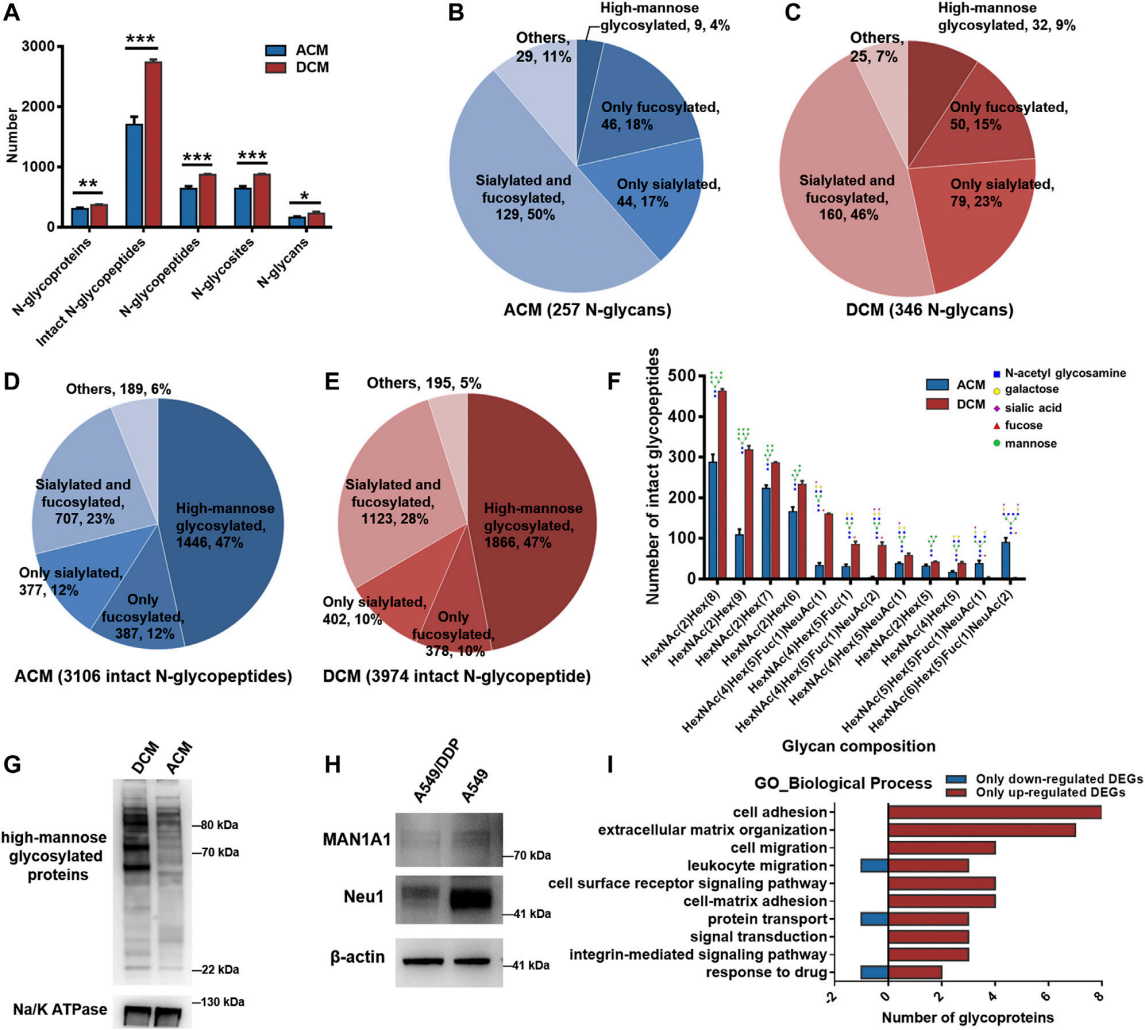
N-glycoproteomic analysis of cell membrane proteins and WB verification of the results. **(A)** Number of identified N-glycoproteins, intact N-glycopeptides, N-glycopeptides, N-glycosites, and N-glycans in ACM and DCM groups. **(B,C)** Number of N-glycans of each type in ACM **(B)** and DCM **(C)** groups. **(D,E)** Number of intact glycopeptides modified with different types of glycans in ACM **(D)** and DCM **(E)** groups. **(F)** Compositions and structures of the top ten glycans detected on the intact glycopeptides from ACM and DCM groups. **(G)** WB detection of global high-mannose glycosylated proteins in ACM and DCM groups. Na/K ATPase was used as an internal standard. **(H)** WB detection of MAN1A1 and Neu1 in A549 and A549/DDP cells. β-actin was used as an internal standard. **(I)** Main biological processes involving the 82 differentially glycosylated proteins and the number of glycoproteins containing only down- or up-regulated glycopeptides implicated in these processes. Error bars represent mean ± SD; * *p* < 0.05, ** *p* < 0.01, *** *p* < 0.001, Student’s *t*-test.

All the experiments were carried out in triplicate for the membrane proteins from each cell line, and the number of identified N-glycoproteins, intact N-glycopeptides, N-glycopeptides, N-glycosites, and N-glycans of each biological replicate were displayed in Supplementary Figure S7. In the ACM group, we identified a total of 3,106 unique intact glycopeptides composed of 257 glycan compositions, 908 glycosites, and 902 unique glycopeptides from 405 glycoproteins. In the DCM group, a total of 3,974 unique intact glycopeptides were identified, which consisted of 346 glycan compositions, 1,079 glycosites, and 1,075 unique glycopeptides from 449 glycoproteins. ACM and DCM groups overlapped across 284 proteins, 1,206 intact glycopeptides, 631 glycopeptides, 633 glycosites and 135 glycan compositions (Supplementary Figure S8). Furthermore, the membrane protein glycosylation were systematically compared between A549 and A549/DDP cells. Evidently, the number of N-glycoproteins, intact N-glycopeptides, N-glycopeptides, N-glycosites, and N-glycans identified in the DCM group were all significantly higher than those in ACM group (Figure 3A), indicating an increase of global glycosylation in the cisplatin-resistant cells. Based on the glycan compositions, the identified N-glycans were classified into five types: high-mannose glycosylated, only fucosylated (but not sialylated), only sialylated (but not fucosylated), fucosylated and sialylated, and others. The dually fucosylated and sialylated type accounted for the highest proportion of diversity (50% for ACM, 46% for DCM) regardless of their abundance, and the high-mannose type contributed only 4 and 9% of diversity, respectively (Figures 3B,C). Moreover, the N-glycan compositions of DCM had larger diversity than those of ACM in high-mannose, fucosylated, or sialylated types of N-glycans. Although the high-mannose type was less diverse, nearly half of glycopeptides were modified with high-mannose glycans for both the ACM and DCM samples (Figures 3D,E). Approximately one third of the glycopeptides were fucosylated or silaylated. Similar to the N-glycans, the number of glycopeptides modified with high-mannose, fucosylated, or sialylated glycans in DCM group were all greater than those in ACM group. In addition, the top ten glycopeptides (the number of peptides containing the same glycan) and the deduced structure of each glycan were shown in Figure 3F. Likewise, the high-mannose glycosylated peptides were proven to have the largest population with those containing HexNAc(2)Hex(8) being the most abundant. These top high-mannose glycosylated peptides were notably increased in A549/DDP cells.

To validate the elevated high-mannose glycosylation in A549/DDP cells revealed by the N-glycoproteomic analysis, the global high-mannose glycosylation level of ACM and DCM groups were detected by WB assay using the lectin Con A as a specific mannose-binding partner. As shown in Figure 3G, the abundance of high-mannose glycosylated proteins was markedly higher in the DCM group, confirming the result of N-glycoproteomic analysis. Furthermore, we examined the expression of two proteins, MAN1A1 and Neu1, which play critical roles in protein glycosylation. MAN1A1 is involved in the maturation of Asn-linked oligosaccharides, and progressively trims α-1,2-linked mannose residues from Man_9_GlcNAc_2_ (Man: mannose, GlcNAc: N-acetylglucosamine) to produce Man_5_GlcNAc_2_ (Ogen-Shtern et al., 2016). WB assay showed that MAN1A1 was down-regulated in A549/DDP cells (Figure 3H). The low expression of MAN1A1 may hamper the processing of N-glycans thereby promoting the accumulation of Man_9_GlcNAc_2_ high-mannose glycosylation in A549/DDP cells, which was also uncovered by the glycoproteomic analysis as peptide modified with the Man_9_GlcNAc_2_ chain [i.e., HexNAc(2)Hex(9)] was one of the most abundant glycopeptides and was markedly increased in A549/DDP cells (Figure 3F). Neu1 functions in sialic acid metabolism and catalyzes the removal of sialic acid moiety from glycoproteins (Bonten et al., 1996). The expression of Neu1 was substantially decreased in the resistant cells (Figure 3H). In view of this, our results suggest that the degradation of sialic acid from glycoproteins is retarded in A549/DDP cells, in turn leading to the accumulation of sialylated glycoproteins in cisplatin-resistant cells.

We also conducted quantitative analysis of the intact N-glycopeptides to detect the abundance changes of glycopeptides between ACM and DCM groups. The DEGs were classified into two types, DEGs with fold changes (DEGs_w_) and DEGs without fold changes (DEGs_w/o_). DEGs_w/o_ included those special glycopeptides that were identified in all the three biological replicates within one group while completely missing in all replicates of the other. Overall nine DEGs_w_ derived from nine glycoproteins were screened out, including six up-regulated (fold change >2.00) and three down-regulated (fold change <0.50) intact glycopeptides (Supplementary Figure S9). The identities and abundance changes of these DEGs_w_ and corresponding proteins were shown in Table 1; Supplementary Figures S10–S18. Besides DEGs_w_, a total of 1,010 DEGs_w/o_ were screened out, among which 833 were exclusively identified in DCM group as up-regulated glycopeptides and 177 were unique in ACM group as down-regulated glycopeptides. Hence, the number of up-regulated glycopeptides was much larger than the down-regulated, confirming the elevated global glycosylation in cisplatin-resistant cells. The top 10 up- and down-regulated DEGs_w/o_ in intensity as well as corresponding glycoproteins were listed in Supplementary Tables S1, S2. Most of the top up-regulated DEGs_w/o_ were derived from cell adhesion molecules, integrins, while the top down-regulated DEGs_w/o_ mainly stemmed from folate receptor.

**TABLE 1:** Identities and abundance changes of the 9 DEGs_w_ (fold change >2.00 or <0.50) and corresponding proteins.

N-glycopeptide	Glycosite	Glycan	Gene name	Intact N-glycopeptide		Protein	
				Fold change	Expression	Fold change	Expression
LPADCIDCTTN#FSCTYGK	87	HexNAc(2)Hex(8)	TM2D3	47.061	Up	NA^a^	NA
DAVNN#ITAK	324	HexNAc(2)Hex(8)	CACNA2D1	6.936	Up	NA	NA
LN#SSTIK	275	HexNAc(5)Hex(6)	LAMP2	6.845	Up	0.613	No change
N#MSFVNDLTVTQDGRK	196	HexNAc(2)Hex(8)	APMAP	5.204	Up	2.982	Up
N#YTADYDK	106	HexNAc(2)Hex(8)	ERLIN1	4.164	Up	3.328	Up
YHYN#GTLLDGTLFDSSYSR	286	HexNAc(2)Hex(9)	FKBP9	2.075	Up	0.627	No change
IAPASN#VSHTVVLRPLK	88	HexNAc(2)Hex(9)	SSR2	0.004	Down	NA	NA
TQN#FTLLVQGSPELK	439	HexNAc(2)Hex(6)	BCAM	0.304	Down	0.249	Down
DTCTQECSYFN#ITK	669	HexNAc(4)Hex(5)Fuc (2)	ITGB1	0.379	Down	3.142	Up

a NA: not quantified in the proteomic analysis.

The 1,019 DEGs including DEGs_w_ and DEGs_w/o_ were derived from 254 glycoproteins, among which 129 were also quantified at the proteomic level. Particularly, we further focused on the differentially glycosylated proteins that contained DEGs but were not changed in protein abundance. A total of 82 differentially glycosylated proteins were screened out containing 54 down-regulated glycopeptides and 302 up-regulated glycopeptides. Specifically, 56 out of 82 glycoproteins contained only the up-regulated glycopeptides while six glycoproteins contained only the down-regulated glycopeptides (Supplementary Figure S19). GO enrichment analysis disclosed that the 82 glycoproteins were primarily implicated in the biological processes of cell adhesion, extracellular matrix (ECM) organization, signal transduction, cell migration, protein transport, and response to drug (Supplementary Figure S20), which are known to be the functions of membrane proteins. Moreover, these biological processes mainly attributed from the glycoproteins containing up-regulated glycopeptides (Figure 3I), suggesting the increased glycosylation of proteins associated with these processes in A549/DDP cells. Additionally, KEGG pathway analysis of the 82 proteins showed that many glycoproteins containing up-regulated DEGs were annotated as cell adhesion molecules (Supplementary Figure S21) and functioned in the ECM-receptor interaction (Supplementary Figure S22), being in good consistency with the findings of GO analysis.

In summary, N-glycoproteomic analysis indicated that the glycosylation of membrane proteins in A549/DDP cells was elevated in terms of the number of identified N-glycoproteins, intact N-glycopeptides, N-glycopeptides, N-glycosites, N-glycans as well as the up-regulated glycopeptides. Moreover, the high-mannose, fucosylated, or sialylated glycosylation levels were increased in the cisplatin-resistant cells. The glycosylation of proteins participating in cell adhesion, ECM organization, signal transduction, cell migration, protein transport, and response to drug was enhanced in the resistant cells, which was closely related to the development of cisplatin resistance.

### Biological Verification of the Results of Proteomic and N-Glycoproteomic Study

Proteomic study of the membrane proteins revealed that the proteins involved in cell adhesion, response to drug, cell migration, and signal transduction were associated with cisplatin resistance. Meanwhile, N-glycoproteomic analysis indicated that the glycosylation of proteins implicated in these biological processes was also altered in the development of cisplatin resistance. Inspired by these findings, we further investigated the cell migration and invasion, as well as the cisplatin uptake and efflux in A549 and A549/DDP cells. In addition, we explored the specific glycosylation type most relevant to cisplatin resistance.

### Cell Migration and Invasion

**FIGURE 4:**
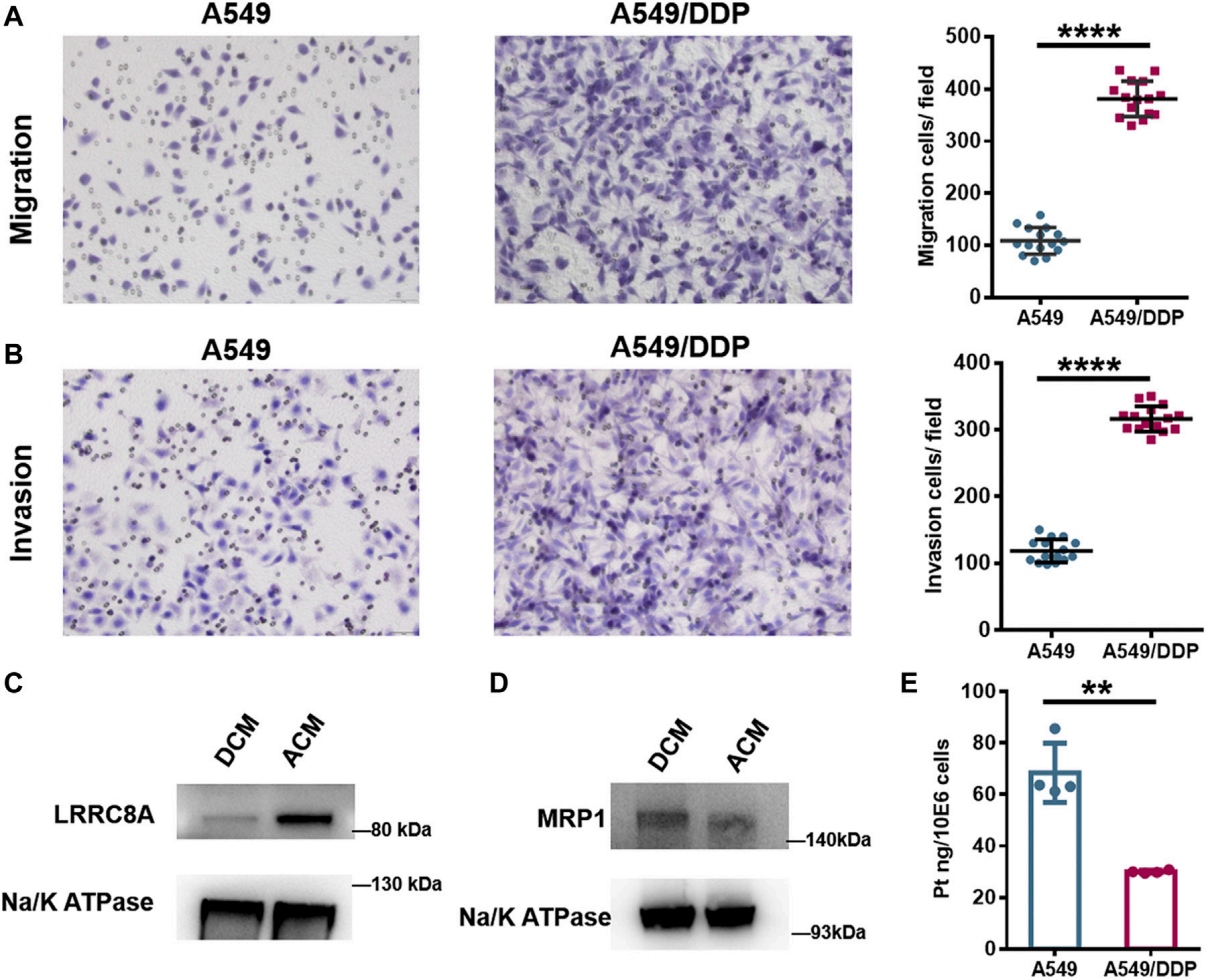
Biological verification of the results of proteomic and N-glycoproteomic study. **(A,B)** Representative images of the Transwell migration **(A)** and invasion assay **(B)** of A549 and A549/DDP cells, and the quantitative number of migration and invasion cells. **(C,D)** WB assay of LRRC8A **(C)** and MRP1 **(D)** in ACM and DCM groups. **(E)** The amount of Pt in A549 and A549/DDP cells measured by ICP-MS. Error bars represent the mean ± SD; ** *p* < 0.01, **** *p* < 0.0001, Student’s *t*-test.

Proteomic analysis showed that the proteins required for cell migration were reduced in the cisplatin-resistant cells, implying the defective migration of A549/DDP cells. Interestingly, the glycosylation of proteins involved in cell migration was increased; for example, the number of identified intact glycopeptides, glycopeptides, glycosites, or glycans of integrin β1 (ITGB1), a key participant in cell adhesion and migration (Leavesley et al., 1993), was increased in DCM group (Supplementary Figures S23A,B), and for most glycosites, the number of modified glycans on each site was also increased (Supplementary Figure S23C). Further assessment using the Transwell system found that the number of migration and invasion A549/DDP cells was much higher than that of A549 cells (Figures 4A,B), indicating that the resistant cells have stronger ability of migration and invasion. This observation revealed that although some proteins involved in the cell migration were down-regulated in the A549/DDP cells, the ability of migration was not impaired and even increased, which was thought to be attributed, at least in part, to the elevated glycosylation of membrane proteins associated with cell adhesion and migration. These results also demonstrate the necessity for integrating proteomic and glycoproteomic detection to comprehensively and accurately study the mechanism of cisplatin resistance.

### Cisplatin Uptake and Efflux

Cell membrane proteins play key roles in the response to drug as there are multiple carriers controlling the uptake and efflux of various drugs. Many carriers have been proposed to function in the accumulation of cisplatin in cells, among which the VRACs are key players. VRACs consist of six LRRC8 subunits (Kefauver et al., 2018). It has been demonstrated that about 50% of cisplatin uptake relies on LRRC8A and LRRC8D under isotonic conditions (Planells-Cases et al., 2015). In the genome-wide screening for Pt drug resistance, loss of LRRC8 subunits of VRACs was found to increase the resistance to cisplatin (Planells-Cases et al., 2015; He et al., 2018). Thus, the LRRC8 subunits play crucial roles in cisplatin accumulation and drug resistance (Planells-Cases et al., 2015; Sørensen et al., 2016; He et al., 2018). Through the quantitative proteomic analysis, we found that LRRC8A was down-regulated in the A549/DDP cells, and WB assay also confirmed the decrease of LRRC8A in the resistant cells (Figure 4C). Moreover, the N-glycoproteomic study showed that the LRRC8A in DCM group was modified with Man_8_GlcNAc_2_ chain at the site of Asn66, whereas no glycosylation was identified in ACM group. Asn66 was located on the extracellular loop of LRRC8A (Supplementary Figure S24A). Molecular modeling showed that the SAA of Asn66 was increased from 21.377 Å^2^ to 29.407 Å^2^ after the modification of Man_8_GlcNAc_2_ oligosaccharide (Supplementary Figures S24B,C). The increased solvent accessibility may affect the channel structure and activity to carry cisplatin into cells (Planells-Cases et al., 2015; Singh et al., 2018). Collectively, the low expression and elevated glycosylation of LRRC8A may cause a reduction in cisplatin uptake by A549/DDP cells, therefore bringing about the resistance to cisplatin.

Apart from the carriers responsible for cisplatin uptake, some transporters mediate the drug efflux out of cells. ATP-binding cassette (ABC) transporters are well-known drug efflux pumps capable of extruding numerous drugs from the cytoplasm (Robey et al., 2018). The ABC superfamily members, multidrug resistance protein 1 (MDR1), MRPs, and breast cancer resistance protein (BCRP), have been proposed to regulate cisplatin sensitivity in various cancer cells, including breast cancer (Yi et al., 2019), lung cancer (Fang et al., 2018; Wu et al., 2019), oesophageal squamous cell carcinoma (Yamasaki et al., 2011), and ovarian carcinoma (Guminski et al., 2006). The role of ABC transporters in cisplatin resistance may vary among the ABC subfamily members and depend on the cancer type. Proteome screening in this study found that MRP1 and MRP4 were slightly over-expressed in A549/DDP cells, and the result of WB assay displayed the same tendency (Figure 4D). Notably, the increased expression of MRP1 and MRP4 has also been reported to correlate with the platinum-based drug resistance in the ovarian carcinoma cells (Beretta et al., 2010). The over-expression of MRP1/4 may increase the export of cisplatin from A549/DDP cells and reduce the intracellular drug concentration. To validate the influence of the alteration of LRRC8A and MRP1/4 on cisplatin accumulation, we used ICP-MS to determine the amount of cisplatin in A549 and A549/DDP cells. For A549 cells, there was 68.5 ng cisplatin in per million cells, while the amount decreased to 30.0 ng for A549/DDP cells (Figure 4E). Thus, the accumulation of cisplatin was significantly reduced in the drug resistant cells due to the low expression and glycosylation of LRRC8A as well as a concomitant increased expression of MRP1/4, thereby conferring cisplatin resistance.

### High-Mannose and Sialylated Glycosylation Contribute to Cisplatin Resistance

N-glycoproteomic analysis indicated the increased glycosylation of membrane proteins in A549/DDP cells. To further determine the specific glycosylation type most relevant to cisplatin resistance, we used several well-established glycosylation inhibitors that could block N-glycan synthesis at different stages to treat the A549 and A549/DDP cells in combination with cisplatin.

**FIGURE 5:**
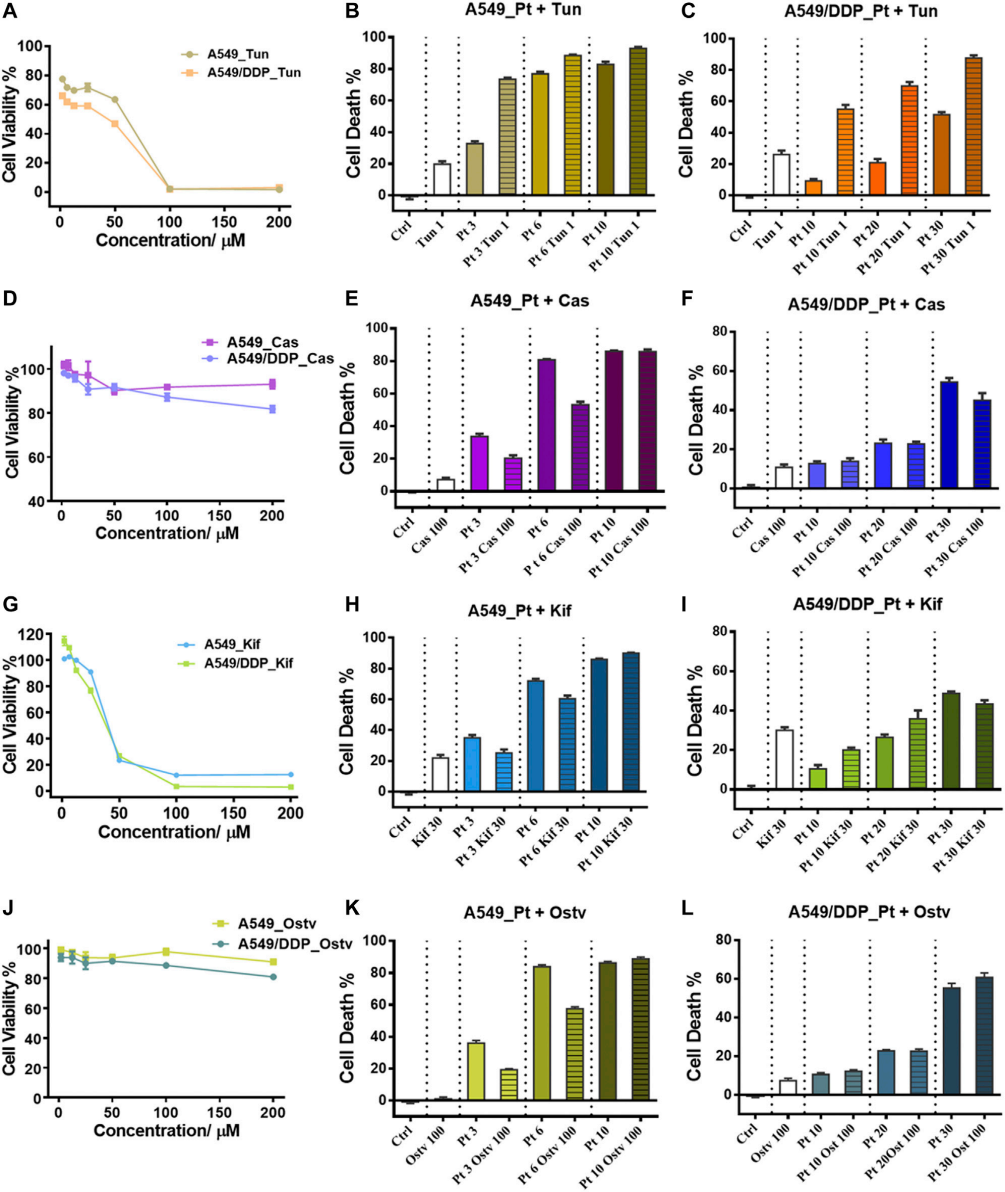
Inhibition of N-glycosylation by small molecule inhibitors. **(A)** Cytotoxicity of Tunicamycin to A549 and A549/DDP cells. **(B,C)** Cytotoxicity of the combination treatment of Tunicamycin (1 μM) and cisplatin to A549 **(B)** and A549/DDP cells **(C)**. **(D)** Cytotoxicity of Castanospermine to A549 and A549/DDP cells. **(E,F)** Cytotoxicity of the combination treatment of Castanospermine (100 μM) and cisplatin to A549 **(E)** and A549/DDP cells **(F)**. **(G)** Cytotoxicity of Kifunensine to A549 and A549/DDP cells. **(H,I)** Cytotoxicity of the combination treatment of Kifunensine (30 μM) and cisplatin to A549 **(H)** and A549/DDP cells **(I)**. **(J)** Cytotoxicity of Oseltamivir to A549 and A549/DDP cells. **(K,L)** Cytotoxicity of the combination treatment of Oseltamivir (100 μM) and cisplatin to A549 **(K)** and A549/DDP cells **(L)**. Concentrations of cisplatin used in the combination treatment are 3, 6 and 9 μM for A549 cells, and 10, 20 and 30 μM for A549/DDP cells. Error bars represent the mean ± SD.

Tunicamycin (Tun), a potent protein glycosylation inhibitor, inhibits protein N-glycosylation entirely at the first step of glycan synthesis (Wu et al., 2018). Tun has been reported to enhance the suppressive effects of cisplatin on cancer cell growth (Noda et al., 1999; Hou et al., 2013; Ahmmed et al., 2019). In this study, we found that Tun exerts cytotoxicity on both A549 and A549/DDP cells with higher toxicity to the resistant cells (Figure 5A). When co-administered with cisplatin, Tun worked synergistically with cisplatin and potentiated its efficacy on A549 and A549/DDP cells, especially at low cisplatin concentrations (Figures 5B,C). The synergistic effect was more apparent on the resistant cells.

Two alkaloids, Castanospermine (Cas) and Kifunensine (Kif), were employed to alter the protein glycosylation in A549 and A549/DDP cells, respectively. Cas and Kif block the trimming reaction of nascent Glc_3_Man_9_GlcNAc_2_ (Glc: glucose) oligosaccharide on glycoproteins by inhibiting the glycosidases involved in the trimming process (Zhong et al., 2020). Cas preferentially inhibits α-glucosidases І and ІІ, causing the accumulation of fully glucosylated high-mannose chains (Pili et al., 1995). Cas did not significantly suppress the proliferation of the 2 cell lines (Figure 5D). In contrast to Tun, Cas increased the tolerance to cisplatin of A549 cells (at the low dosage of cisplatin) and A549/DDP cells (at the high dosage of cisplatin) as the cell death of combinatorial administration was lower than that of individual treatment with cisplatin (Figures 5E,F). Kif selectively inhibits the α-mannosidase І, resulting in the accumulation of Man_7-9_GlcNAc_2_ high-mannose type oligosaccharides on glycoproteins (Zhou et al., 2008), which were also the top three abundant glycans identified in ACM and DCM groups (Figure 3F). Although being cytotoxic to A549 and A549/DDP cells (Figure 5G), Kif also increased the cell survival in a similar way to Cas when working in combination with cisplatin (Figures 5H,I).

Oseltamivir phosphate (Ostv) is an efficient small-molecule inhibitor of neuraminidase that prevents the cleavage of sialic acid from glycan chains and increases the level of sialylated glycosylation (de Oliveira et al., 2015). Ostv alone did not have a strong suppressive effect on cell proliferation (Figure 5J). When treated with a combination of Ostv and cisplatin, A549/DDP cells showed no significant difference in cell death from those treated with cisplatin alone, while A549 cells survived more compared with the individual treatment (Figures 5K,L).

Collectively, these findings suggest that inhibition of protein N-glycosylation could enhance the anticancer activity of cisplatin on both A549 and A549/DDP cells. However, promoting the high-mannose or sialylated type of glycosylation attenuated the sensitivity of A549 cells to cisplatin. Therefore, the increased high-mannose or sialylated glycosylation play critical roles in the development of cisplatin resistance and contribute to the resistant phenotype.

## Conclusion

In this work, we have integrated proteomic and N-glycoproteomic detection for the comprehensive characterization of the membrane proteins associated with cisplatin resistance in non-small cell lung cancer cells. Based on this method, we found that the proteins involved in cell adhesion, cell migration, response to drug, and signal transduction were significantly altered in abundance and glycosylation in the development of cisplatin resistance. Consequently, the ability of migration and invasion was substantially increased in cisplatin-resistant cells, further aggravating their malignancy. On the other hand, the concentration of cisplatin was significantly reduced in the resistant cells, concomitant with the down-regulation of LRRC8A and over-expression of MRP1 and MRP4, thereby decreasing the amount of cellular cisplatin and conferring the resistance. In addition, the global glycosylation of membrane proteins was significantly elevated in the cisplatin-resistant cells, and inhibition of protein N-glycosylation potentiated the anticancer efficacy of cisplatin. Particularly, increasing the high-mannose or sialylated type of glycosylation promoted the cell resistance to cisplatin, revealing the key glycosylation type associated with cisplatin-resistance. These results give new insights into the mechanism of cisplatin resistance. It is also worth noting that the integrated method is versatile and highly efficient for obtaining multilevel and comprehensive information on the expression and glycosylation of proteins in a single experiment. We anticipate its broad application in the study on the mechanisms of drug resistance.

## Data Availability

The datasets presented in this study can be found in online repositories. The names of the repository/repositories and accession number(s) can be found below: http://www.proteomexchange.org/, PXD028691.
