# Supplementary material for: Elevated N-Glycosylation Contributes to the Cisplatin Resistance of Non-Small Cell Lung Cancer Cells Revealed by Membrane Proteomic and Glycoproteomic Analysis
Source: Front Pharmacol. 2021 Dec 22;12:805499. doi: 10.3389/fphar.2021.805499 (PMC8728018; doi:10.3389/fphar.2021.805499)
Supplement: Supplementary file 2 [file DataSheet1.docx]

**Elevated N-glycosylation Contributes to the Cisplatin Resistance of Non-small Cell Lung Cancer Cells Revealed by Membrane Proteomic and Glycoproteomic Analysis**

Wenjuan Zeng^1^, Shanshan Zheng^1^, Yonghong Mao^2^, Shisheng Wang^1^, Yi Zhong^1^, Wei Cao^1^, Tao Su^1^, Meng Gong^1^, Jingqiu Cheng^1^, Yong Zhang^1,3*^, Hao Yang^1,3*^

^1^NHC Key Laboratory of Transplant Engineering and Immunology, Institutes for Systems Genetics; National Clinical Research Center for Geriatrics, West China Hospital, Sichuan University, Chengdu 610041, China.

^2^Institute of Thoracic Oncology, West China Hospital, Sichuan University, Chengdu 610041, China.

^3^Sichuan Provincial Engineering Laboratory of Pathology in Clinical Application, West China Hospital, Sichuan University, Chengdu 610041, China.

***Corresponding Authors:** Hao Yang, PhD, Associate Professor, Key Laboratory of Transplant Engineering and Immunology, West China Hospital, Sichuan University

Yong Zhang, PhD, Assistant Professor, Key Laboratory of Transplant Engineering and Immunology, West China Hospital, Sichuan University

**Address:** No. 1, Keyuan 4th Road, Gaopeng Avenue, Hi-tech Zone, Chengdu 610041, China. Phone: +86-28-85164031; Fax: +86-28-85164031; E-mail: yanghao@scu.edu.cn; nankai1989@foxmail.com


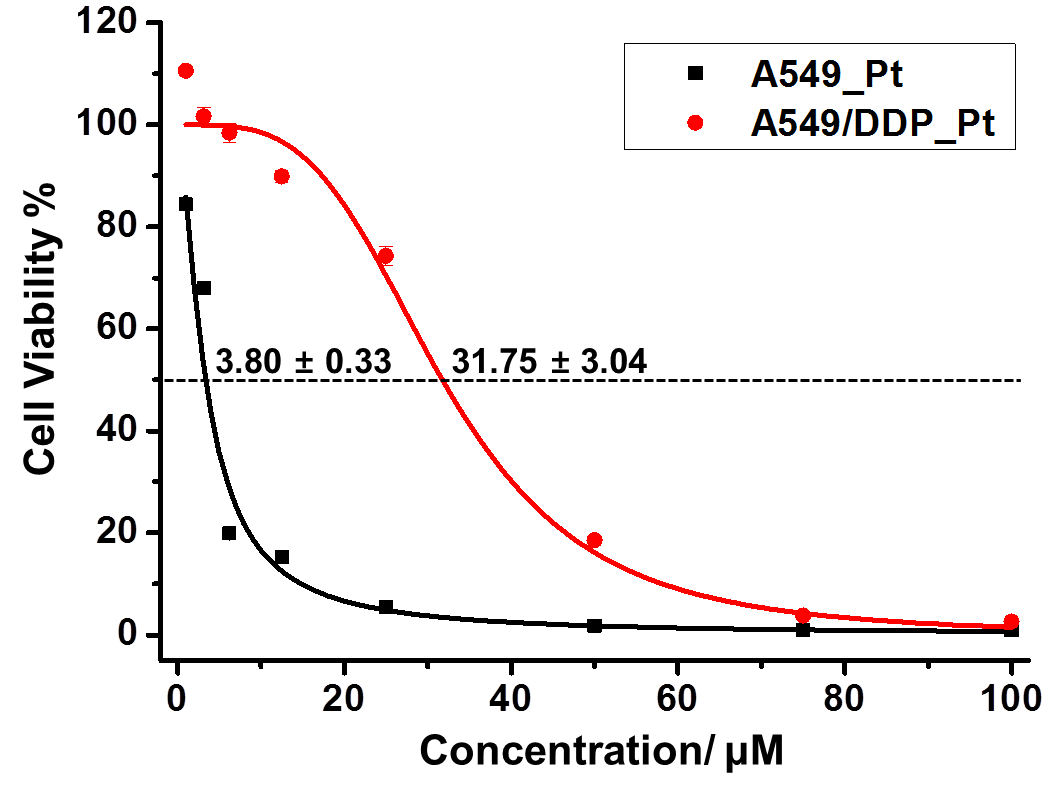


**Figure S1.** Cytotoxicity of cisplatin to A549 (black) and A549/DDP cells (red) measured by MTT assay. The half maximal inhibitory concentration (IC_50_) of cisplatin were 3.80 ± 0.33 μM for A549 cells and 31.75 ± 3.04 μM for A549/DDP cells calculated from the fitted dose-dependent curve, so the resistance factor was 8.36.


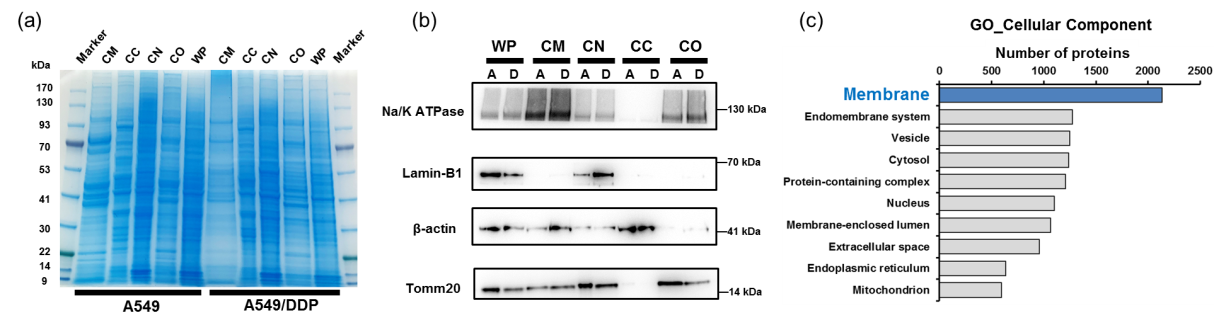


**Figure S2.** Characterization of the purity of the cell membrane proteins. (a) SDS-PAGE analysis of the proteins extracted from cell membrane (CM), cytosol (CC), nucleus (CN), organelle (CO), and the whole cell lysate (WP) of A549 and A549/DDP cells. (b) Western blot analysis of proteins from WP, CM, CN, CC, and CO of A549 (A) and A549/DDP (D) cells. The marker for CM, CN, CC and CO were Na/K ATPase, Lamin-B1, β-actin and Tomm20, respectively. (c) GO analysis of the enriched cellular component of the identified 2906 proteins.


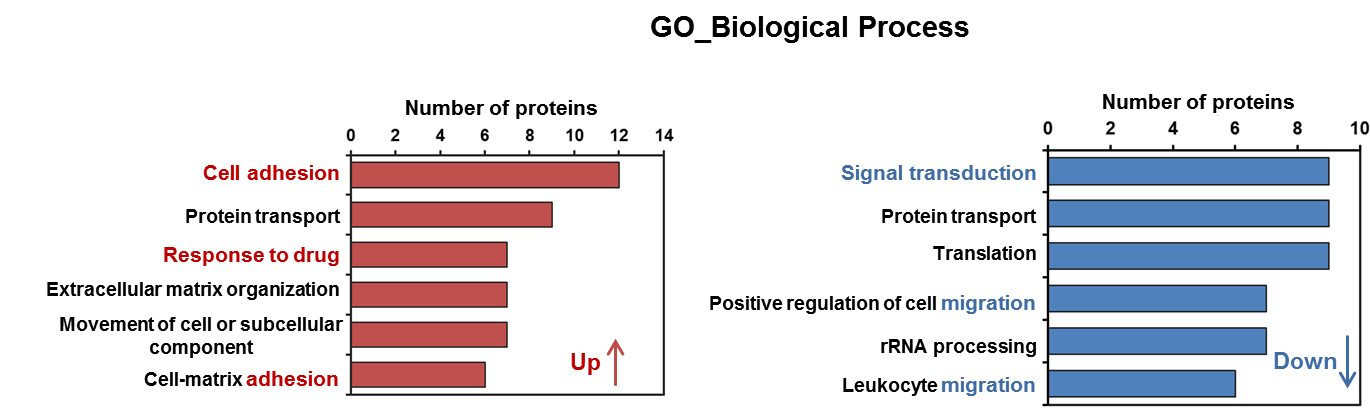


**Figure S3.** GO analysis of the mainly enriched biological processes of the up-regulated proteins (left) and down-regulated proteins (right).


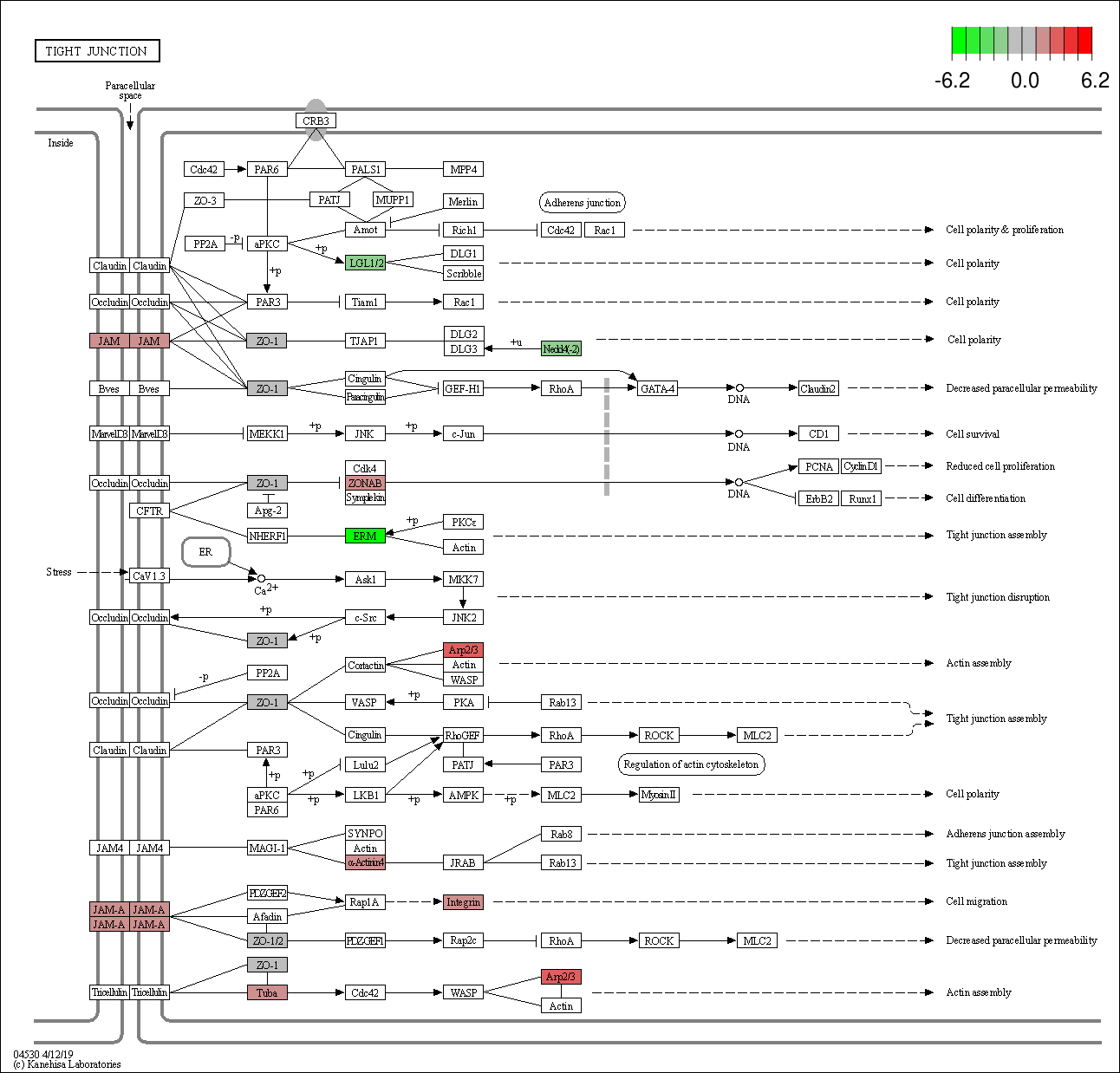


Fold change

**Figure S4.** KEGG pathway of tight junction. Red nodes: up-regulated proteins; Green nodes: down-regulated proteins; Grey nodes: proteins with no significant abundance change; White nodes: proteins not identified.


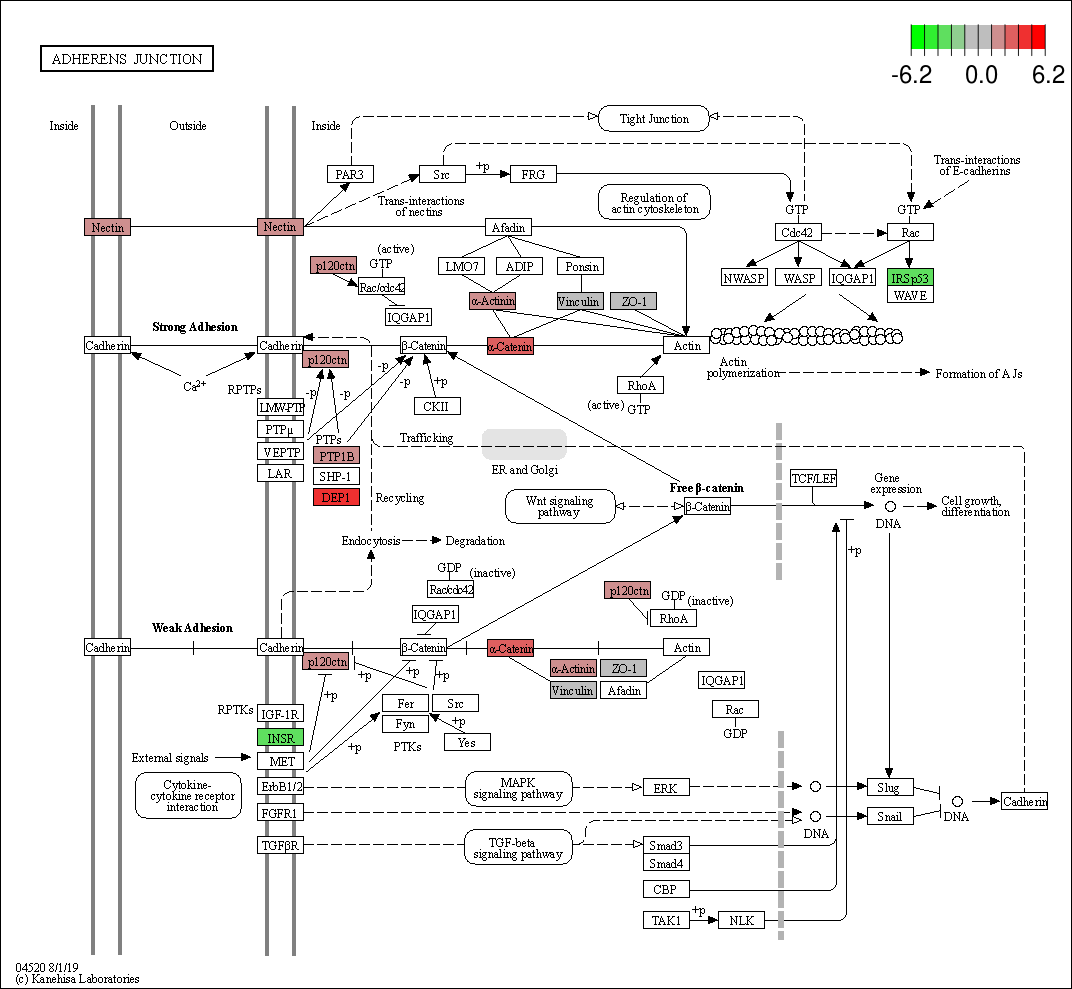


Fold change

**Figure S5.** KEGG pathway of adherens junction. Red nodes: up-regulated proteins; Green nodes: down-regulated proteins; Grey nodes: proteins with no significant abundance change; White nodes: proteins not identified.


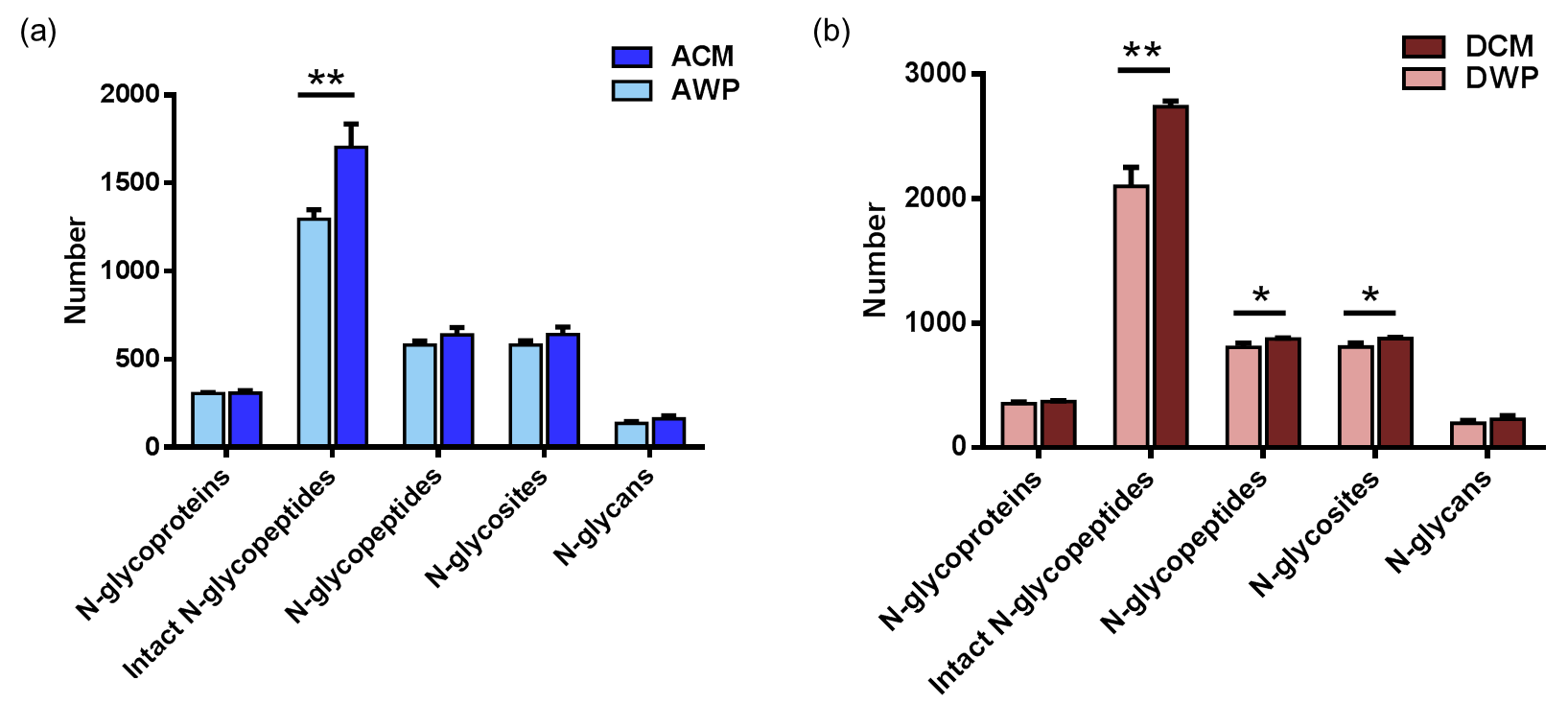


**Figure S6**. Comprehensive comparison between ACM and AWP (a) or DCM and DWP (b) groups in the number of identified N-glycoproteins, intact N-glycopeptides, N-glycopeptides (peptide backbones without glycans), N-glycosites and N-glycans. Error bars represent mean ± SD; ******P*< 0.05, *******P*< 0.01, Student’s *t*-test.


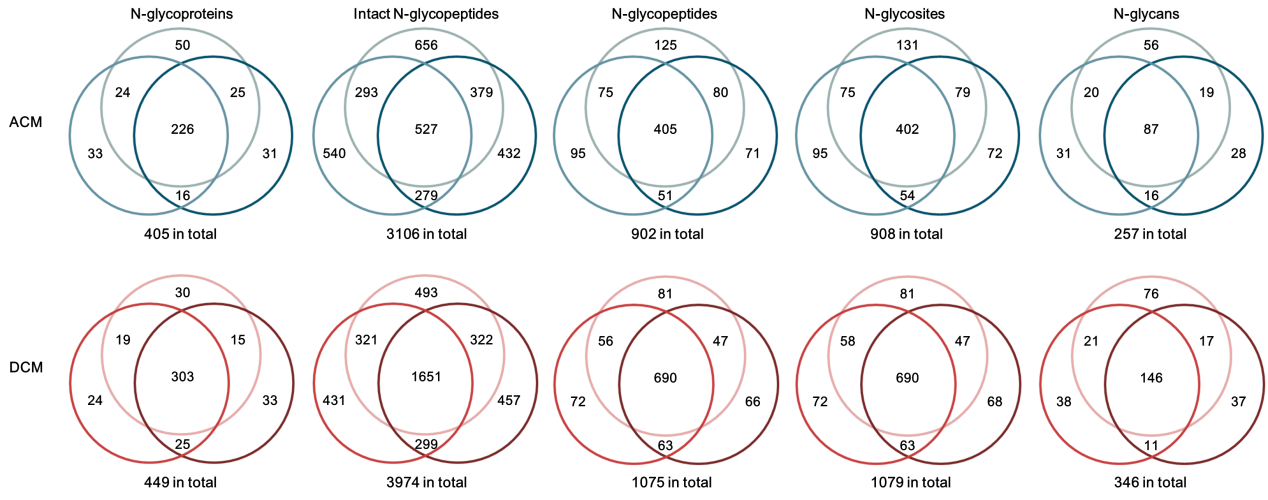


**Figure S7.** Venn diagrams of the N-glycoproteins, intact N-glycopeptides, N-glycopeptides, N-glycosites and N-glycans identified in the three biological replicates of ACM (top) and DCM (bottom) group.


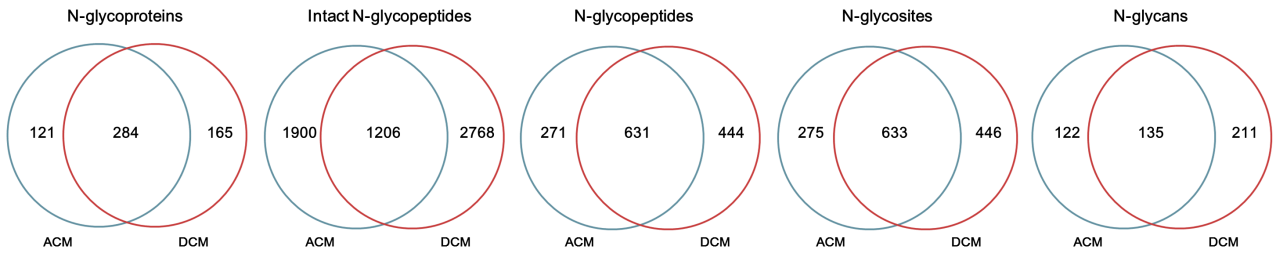


**Figure S8.** Venn diagrams of all the identified N-glycoproteins, intact N-glycopeptides, N-glycopeptides, N-glycosites and N-glycans in ACM (blue) and DCM (red) groups.


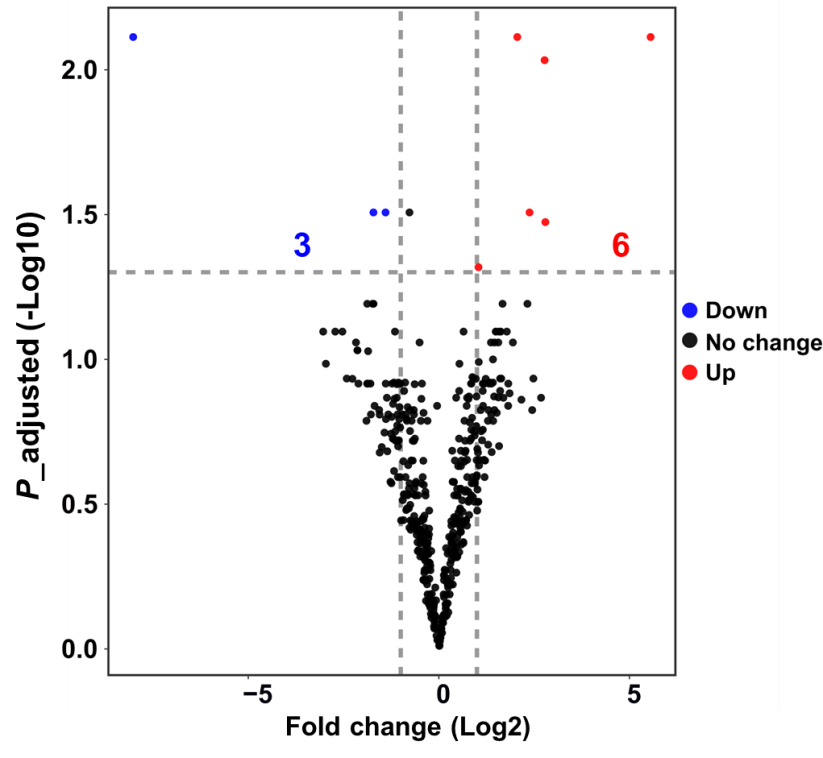


**Figure S9.** Volcano plot of quantitative N-glycoproteomic data constructed using fold changes (DCM/ACM) and adjusted *P* values. Red dots: significantly up-regulated intact N-glycopeptides (fold change > 2.00, *P* < 0.05). Blue dots: significantly down-regulated intact N-glycopeptides (fold change < 0.50, *P* < 0.05). Black dots: intact N-glycopeptides with no significant changes.


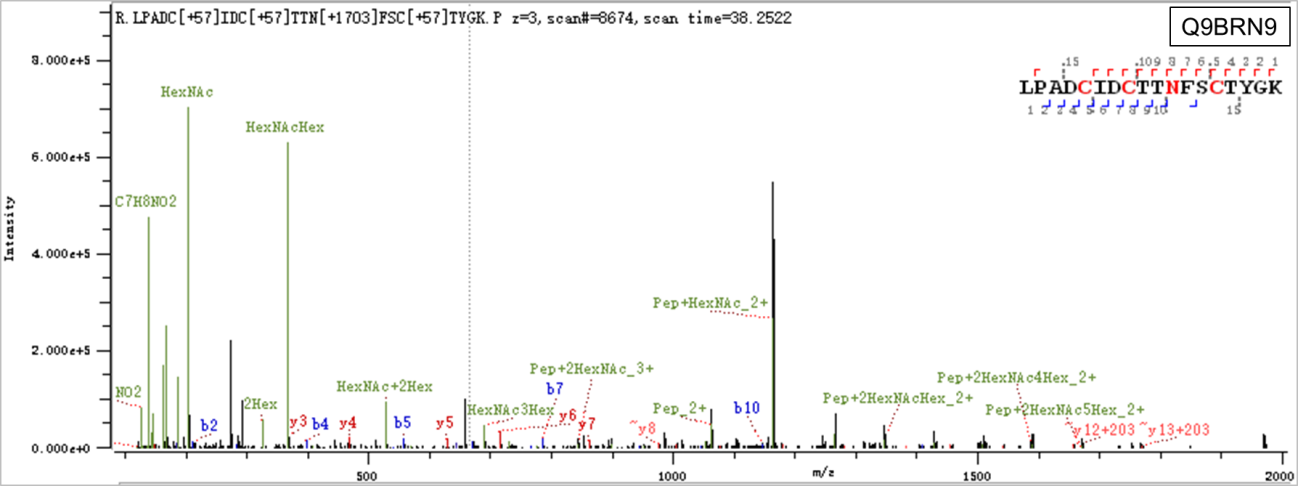


**Figure S10.** Annotated MS/MS spectrum of the intact N-glycopeptide LPADC[+57.021]IDC[+57.021]TTN[+1702.581]FSC[+57.021]TYGK of TM2 domain-containing protein 3 (Q9BRN9).


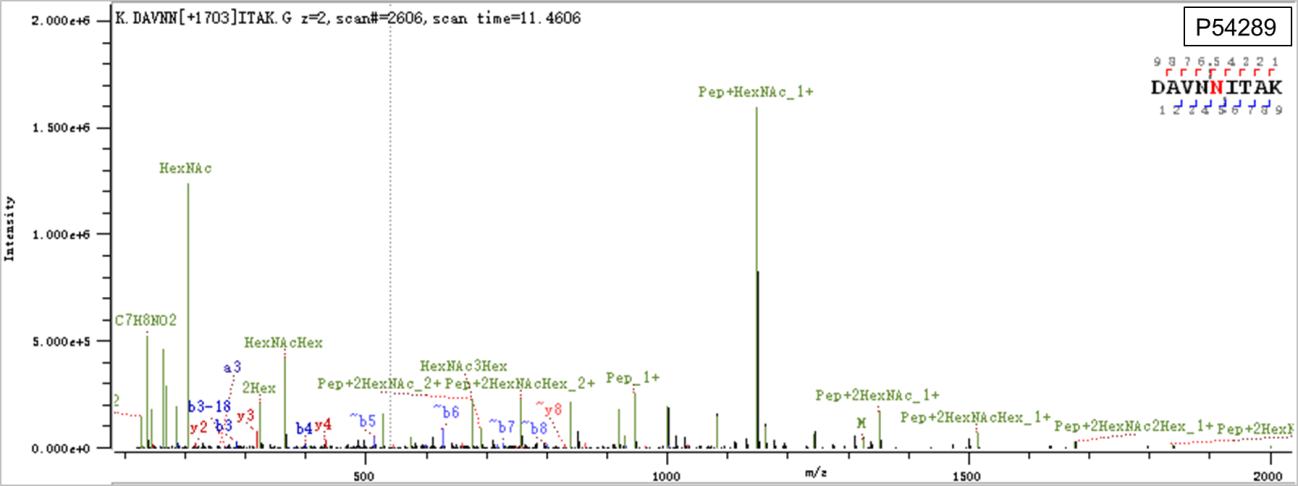


**Figure S11.** Annotated MS/MS spectrum of the intact N-glycopeptide DAVNN[+1702.581]ITAK of Voltage-dependent calcium channel subunit alpha-2/delta-1 (P54289).


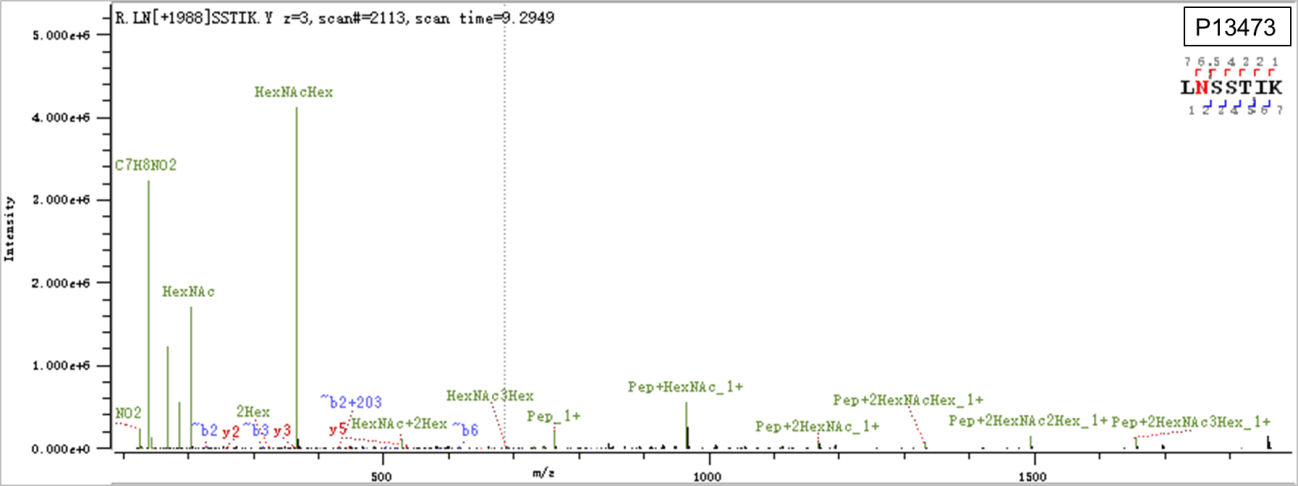


**Figure S12.** Annotated MS/MS spectrum of the intact N-glycopeptide LN[+1987.714]SSTIK of Lysosome-associated membrane glycoprotein 2 (P13473).


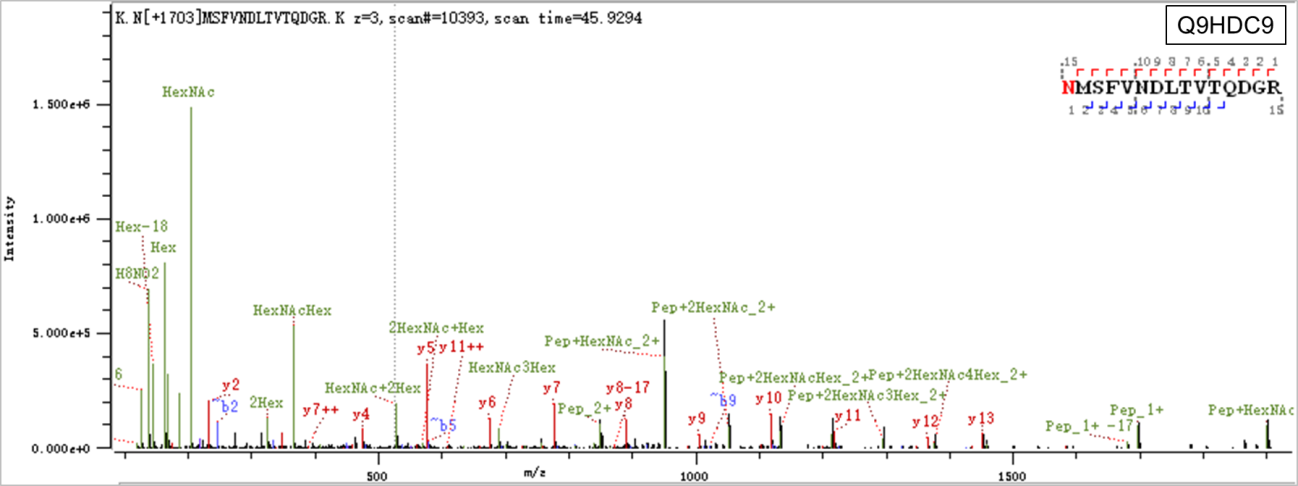


**Figure S13.** Annotated MS/MS spectrum of the intact N-glycopeptide N[+1702.581]MSFVNDLTVTQDGRK of Adipocyte plasma membrane-associated protein (Q9HDC9).


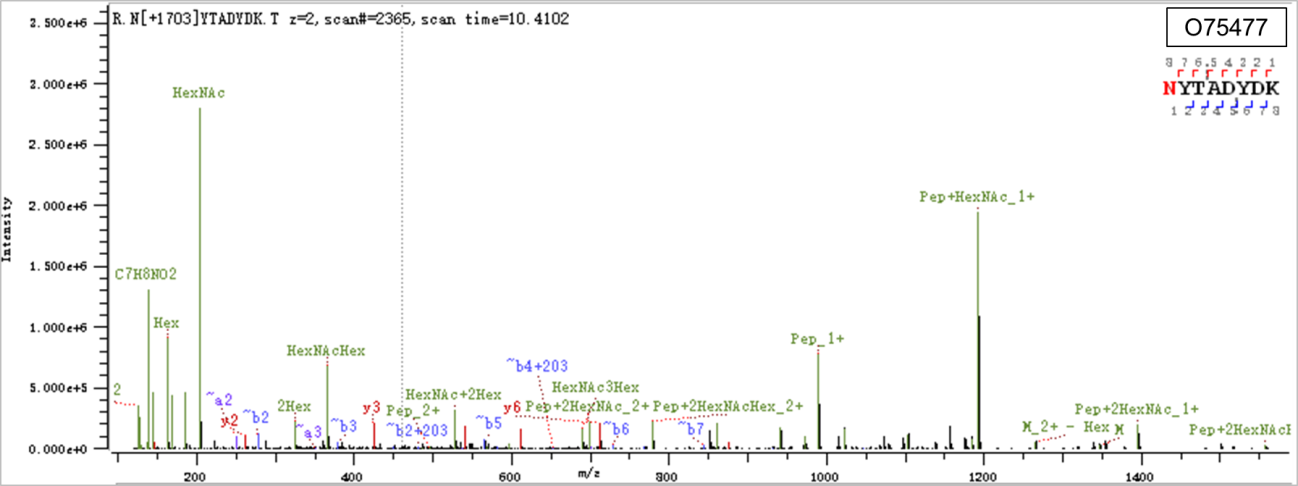


**Figure S14.** Annotated MS/MS spectrum of the intact N-glycopeptide N[+1702.581]YTADYDK of Erlin-1 (O75477).


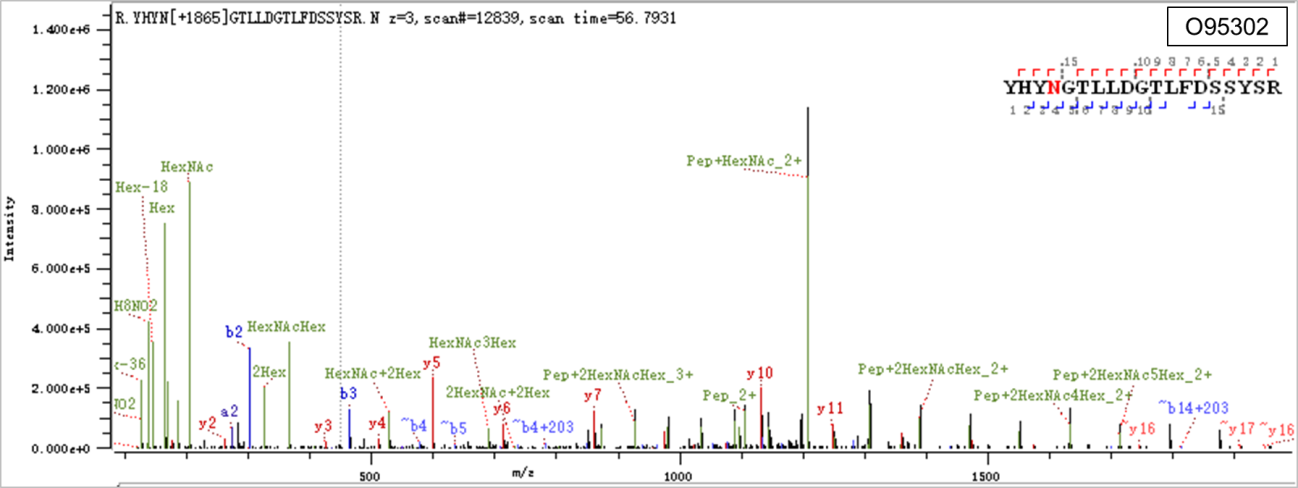


**Figure S15.** Annotated MS/MS spectrum of the intact N-glycopeptide YHYN[+1864.634]GTLLDGTLFDSSYSR of Peptidyl-prolyl cis-trans isomerase (O95302).


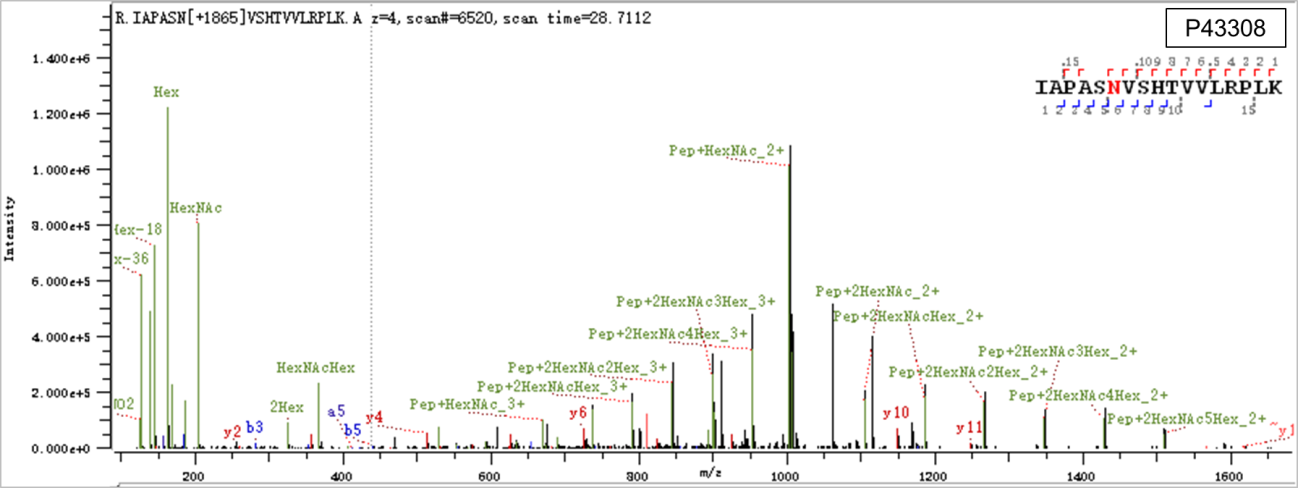


**Figure S16.** Annotated MS/MS spectrum of the intact N-glycopeptide IAPASN[+1864.634]VSHTVVLRPLK of Translocon-associated protein subunit beta (P43308).


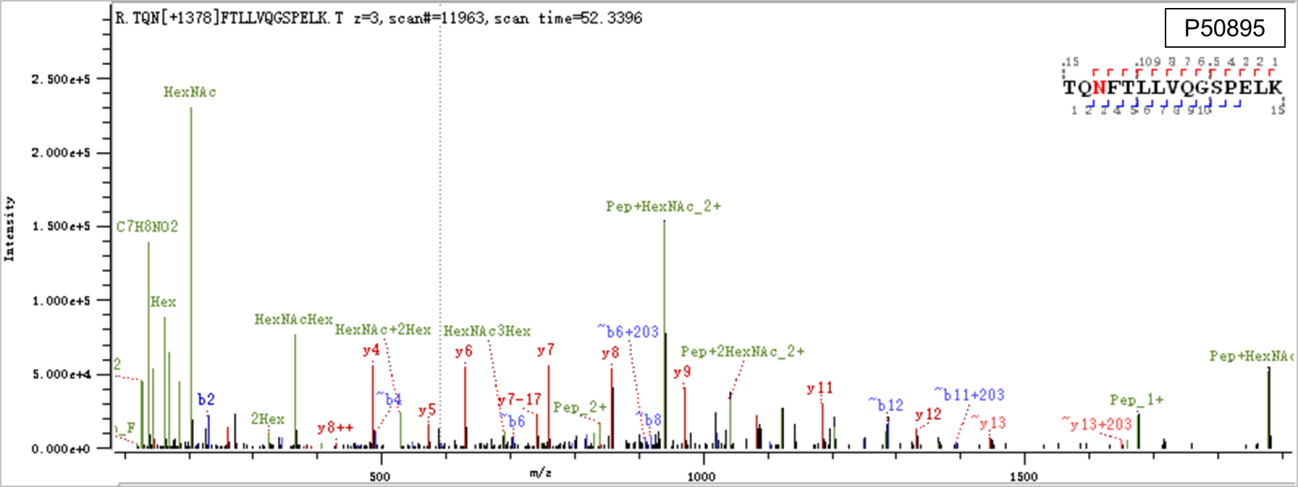


**Figure S17.** Annotated MS/MS spectrum of the intact N-glycopeptide TQN[+1378.476]FTLLVQGSPELK of Basal cell adhesion molecule (P50895).


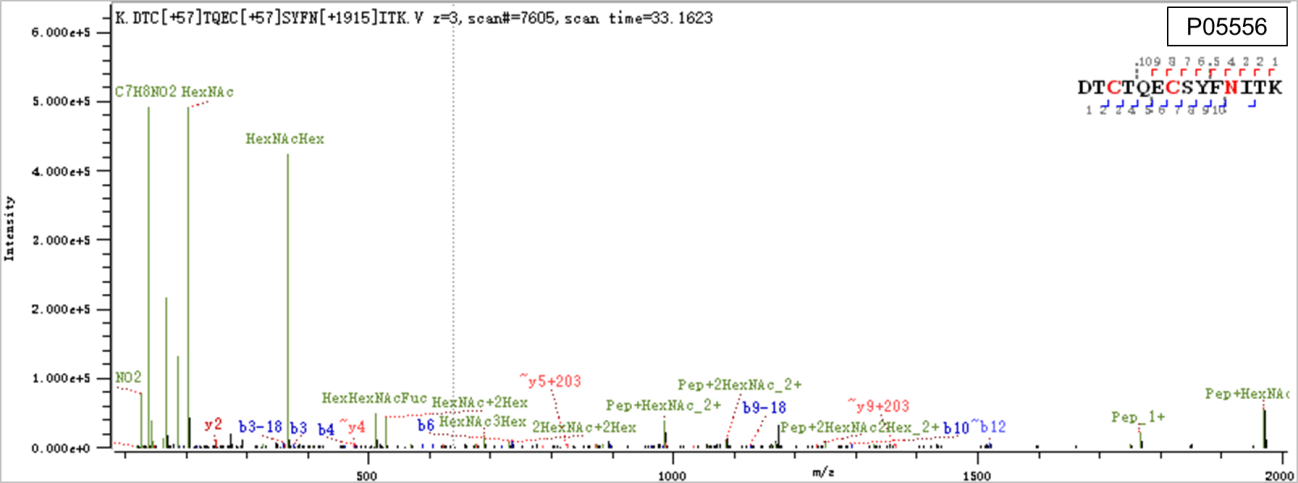


**Figure S18.** Annotated MS/MS spectrum of the intact N-glycopeptide DTC[+57.021]TQEC[+57.021]SYFN[+1914.697]ITK of Integrin beta-1 (P05556).

**Table S1.** Top 10 up-regualted DEGs_w/o_ in intensity.


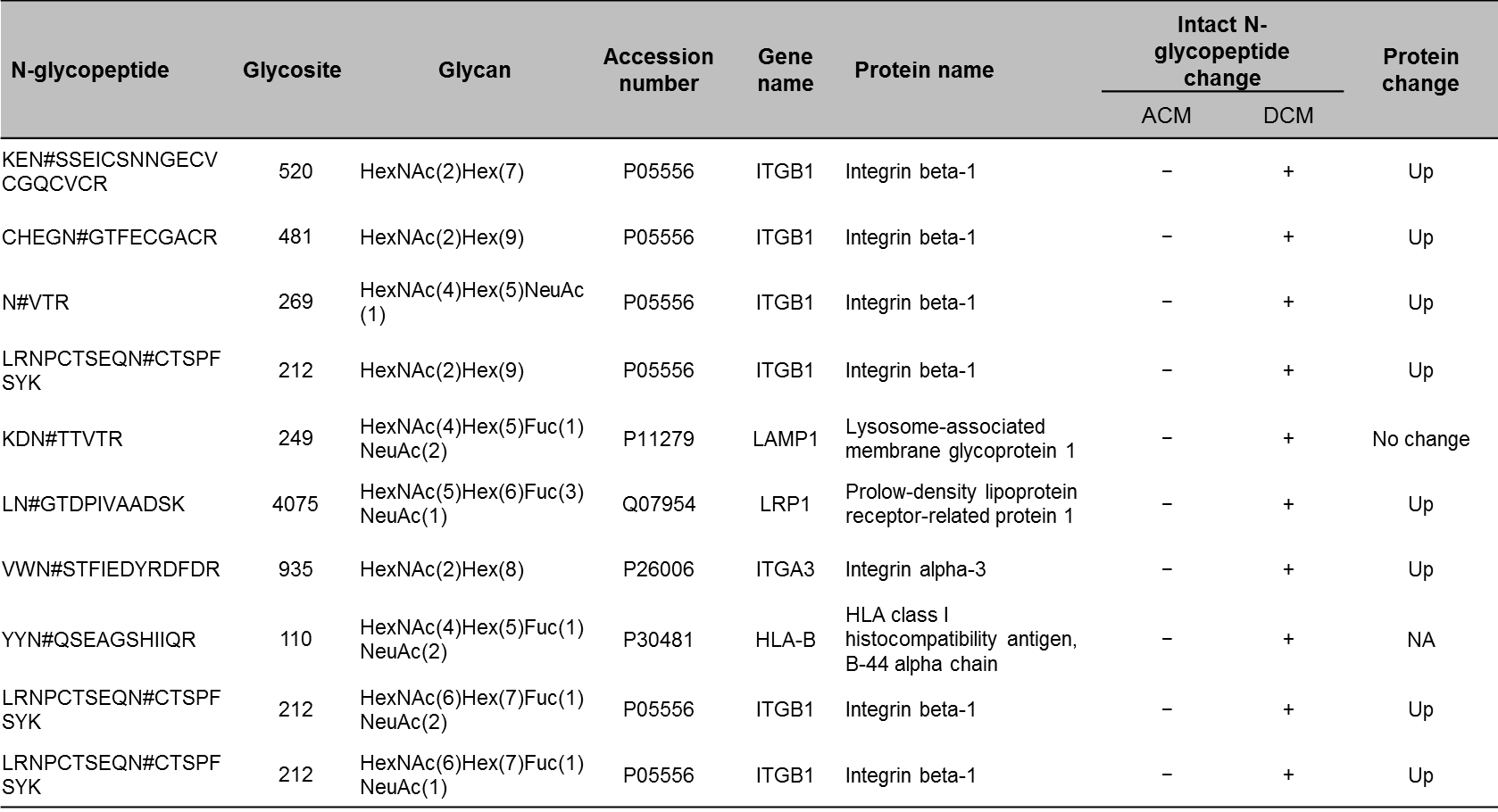


“−”: unidentified; “+”: identified; “NA”: not quantified in proteomic analysis.

**Table S2.** Top 10 down-regualted DEGs_w/o_ in intensity.


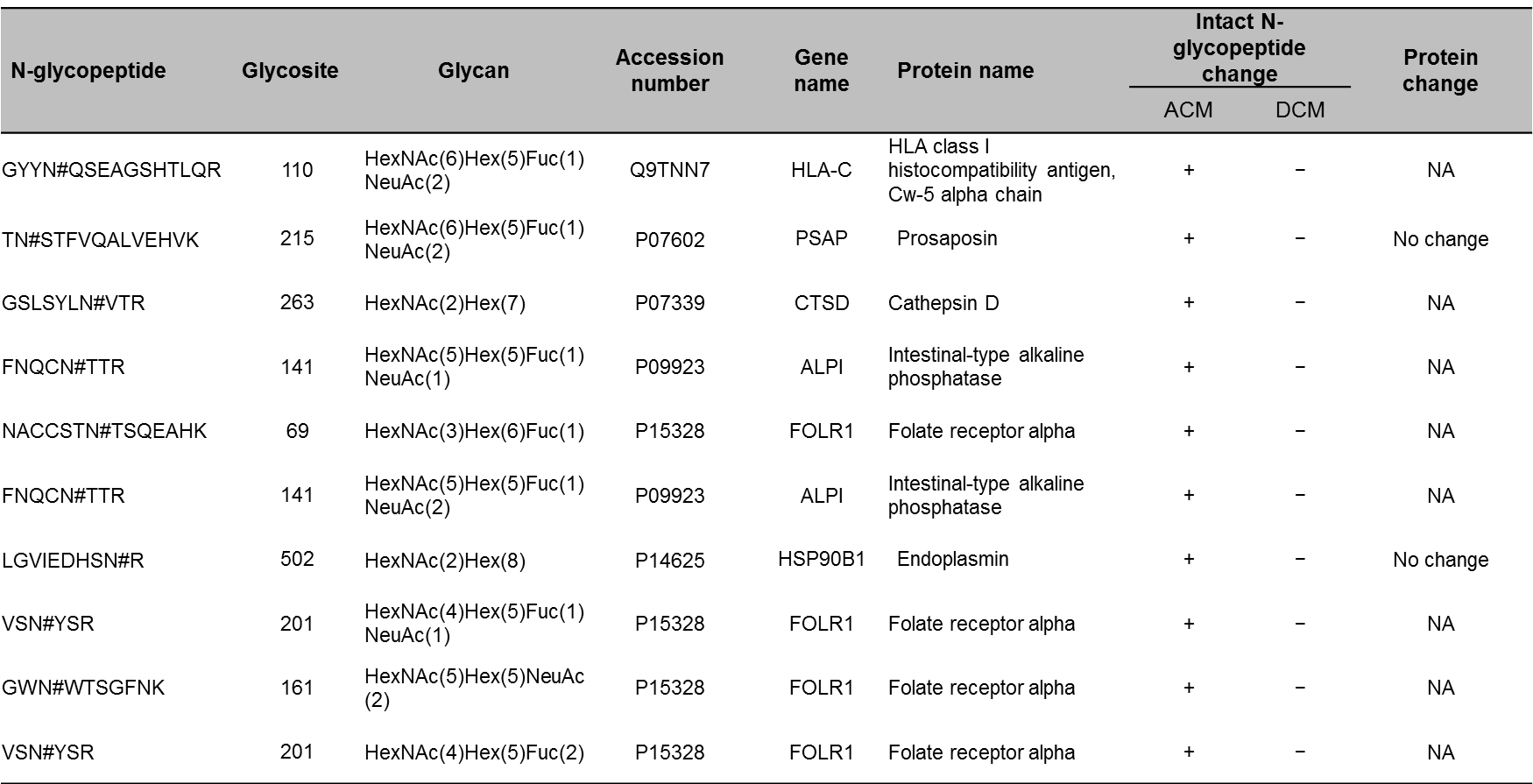


“−”: unidentified; “+”: identified; “NA”: not quantified in proteomic analysis.


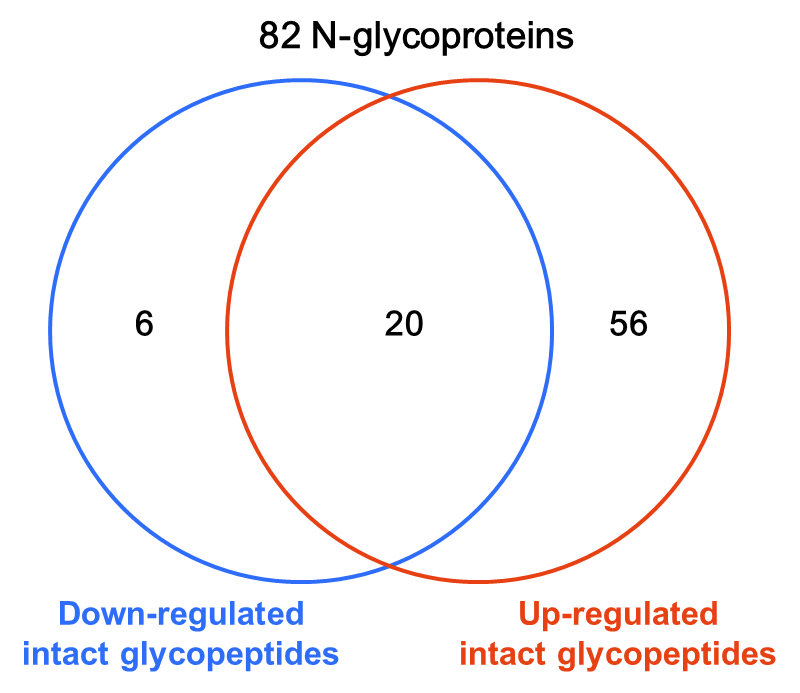


**Figure S19.** Venn diagram of the 82 N-glycoproteins containing up- (red) or down-regulated (blue) intact N-glycopeptides whereas the protein abundance was not changed. Six out of the 82 glycoproteins contained only the down-regulated glycopeptides, while 56 glycoproteins contained only the up-regulated glycopeptides.


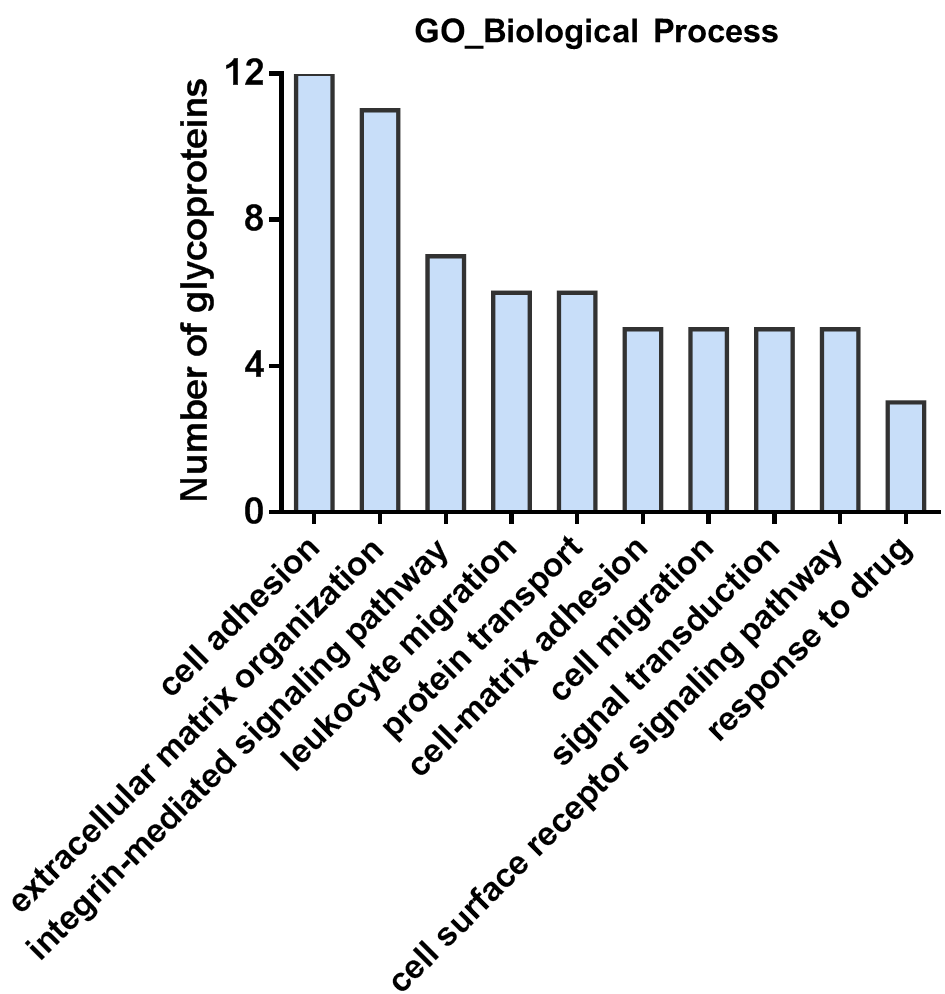


**Figure S20.** GO analysis of the mainly enriched biological processes of the 82 differentially glycosylated proteins.


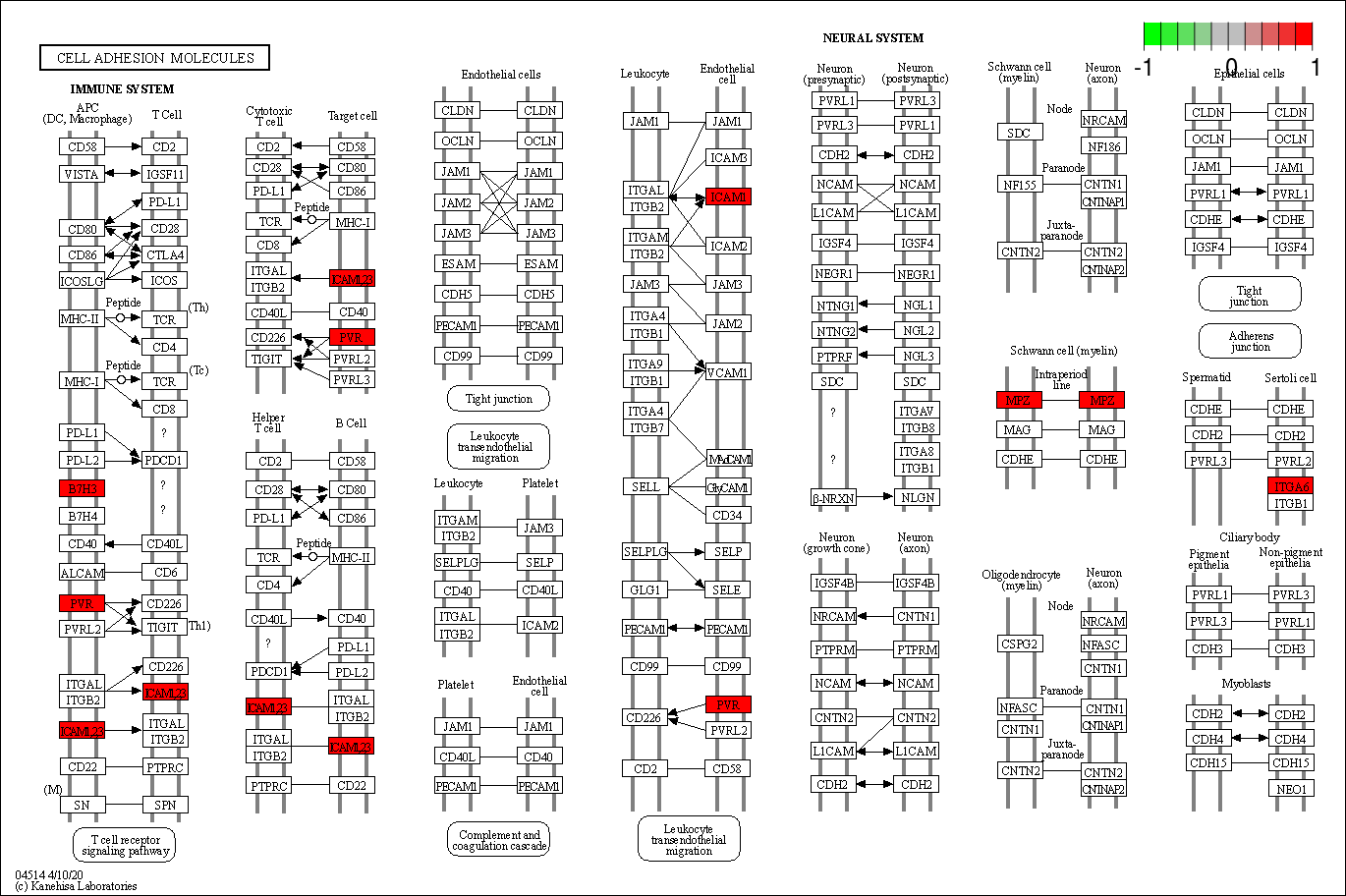


**Figure S21.** KEGG pathway of cell adhesion molecules. Red nodes: the glycoproteins containing only up-regulated glycopeptides; White nodes: proteins not identified in the 82 differentially glycosylated proteins.


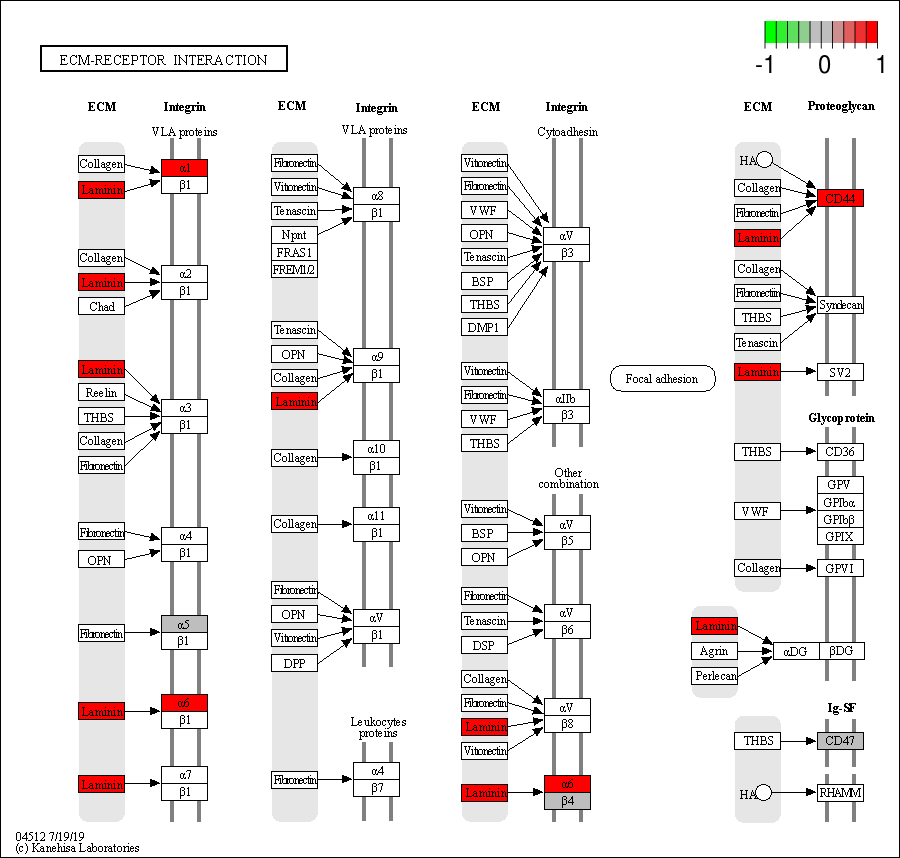


**Figure S22.** KEGG pathway of ECM-receptor interaction. Red nodes: the glycoproteins containing only up-regulated glycopeptides; Grey nodes: the glycoproteins containing both down- and up-regulated glycopeptides; White nodes: proteins not identified in the 82 differentially glycosylated proteins.


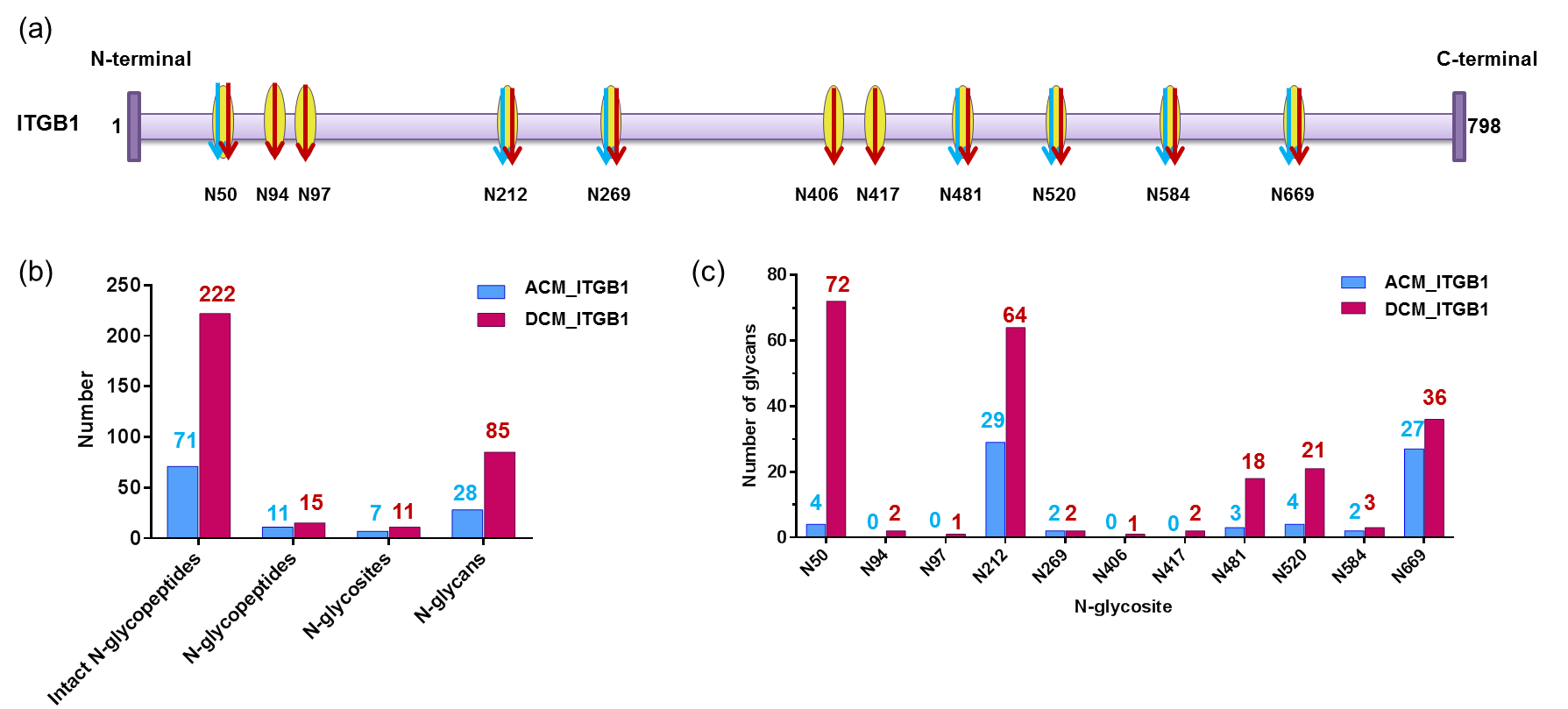


**Figure S23.** The glycosylation of integrin β1 (ITGB1). (a) The distribution of the glycosites (yellow oval) on the sequence of ITGB1 identified in ACM (blue arrow) or DCM (red arrow) group. (b) The number of intact N-glycopeptides, N-glycopeptides, N-glycosites and N-glycans identified in ACM and DCM group. (c) The number of N-glycans in each glycosite identified in ACM and DCM group.


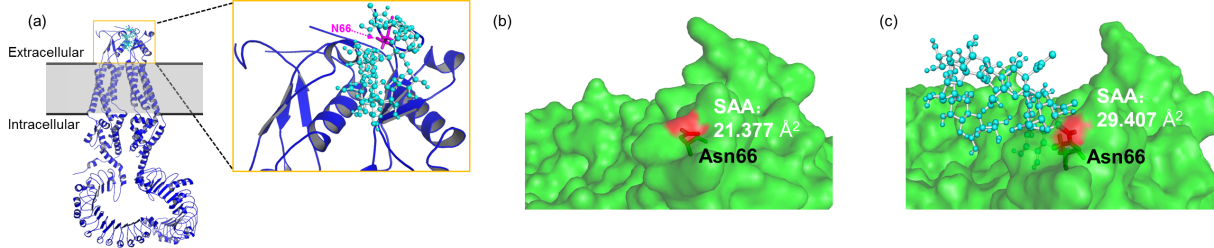


**Figure S24.** Molecular modeling. (a) The molecular model of the glycosylated human volume regulated anion channel protein (VRAC). The glycosite Asn66 (magenta) modified with the Man_8_GlcNAc_2_ oligosaccharide (cyan) was located on the extracellular loop of the LRRC8A subunit of VRAC protein (blue, PDB: 6DJB). For better visualization, only the two symmetrical subunits of the hexamer were shown. (b, c) Solvent accessible areas (SAA, Å^2^) of the NH_2_ group on the sidechain of Asn66 residue before (b) and after (c) the glycosylation calculated using the VEGA ZZ module of PyMOL program.
